# A Parzen window-based approach for the detection of locally enriched transcription factor binding sites

**DOI:** 10.1186/1471-2105-14-26

**Published:** 2013-01-21

**Authors:** Alexis Vandenbon, Yutaro Kumagai, Shunsuke Teraguchi, Karlou Mar Amada, Shizuo Akira, Daron M Standley

**Affiliations:** 1 Laboratory of Systems Immunology, Immunology Frontier Research Center, Osaka University, Osaka, Japan; 2 Laboratory of Host Defense, Immunology Frontier Research Center, Osaka University, Osaka, Japan; 3 Department of Host Defense, Research Institute for Microbial Diseases, Osaka University, Osaka, Japan

**Keywords:** Regulation of transcription, Promoter sequence, Transcription factor binding site, Parzen window

## Abstract

**Background:**

Identification of *cis*- and *trans*-acting factors regulating gene expression remains an important problem in biology. Bioinformatics analyses of regulatory regions are hampered by several difficulties. One is that binding sites for regulatory proteins are often not significantly over-represented in the set of DNA sequences of interest, because of high levels of false positive predictions, and because of positional restrictions on functional binding sites with regard to the transcription start site.

**Results:**

We have developed a novel method for the detection of regulatory motifs based on their local over-representation in sets of regulatory regions. The method makes use of a Parzen window-based approach for scoring local enrichment, and during evaluation of significance it takes into account GC content of sequences. We show that the accuracy of our method compares favourably to that of other methods, and that our method is capable of detecting not only generally over-represented regulatory motifs, but also locally over-represented motifs that are often missed by standard motif detection approaches. Using a number of examples we illustrate the validity of our approach and suggest applications, such as the analysis of weaker binding sites.

**Conclusions:**

Our approach can be used to suggest testable hypotheses for wet-lab experiments. It has potential for future analyses, such as the prediction of weaker binding sites. An online application of our approach, called LocaMo Finder (Local Motif Finder), is available at http://sysimm.ifrec.osaka-u.ac.jp/tfbs/locamo/.

## Background

Regulation of transcription in eukaryote cells is controlled by the binding of transcription factors (TFs) to specific binding sites in the regulatory regions of their target genes. In this way, transcription factor binding sites (TFBSs) play an important role in the regulation of gene expression. Unfortunately, TFBSs are hard to identify; experimental approaches are laborious and costly, and computational analyses are plagued by high false positive rates. TFBSs are extremely short compared to the regions in which they are present, and TFs typically bind to a variety of motifs.

One of the many difficulties faced by TFBS detection approaches is that some TFBSs are restricted in their location with regard to the transcription start site (TSS). An extreme example of such a motif is the TATA-box, which is positioned about 25 to 30 bases upstream of the TSS. However, computational analyses usually use sequences of a fixed length (for eukaryotes typically 1000 bps or longer). In such cases, the region in which genuine regulatory motifs are positioned is small compared to the input sequence length, which makes position-restricted TFBSs hard to detect using standard approaches.

A number of studies have investigated the positional tendencies of nucleotide motifs and TFBSs in genome-wide or large sets (>1000 sequences) of promoter sequences [1-5]. Approaches aiming at predicting locally enriched TFBSs in smaller sets of sequences have also been reported. Many of these approaches involve counting the number of TFBSs in windows within the regulatory regions, either using a sliding window approach [6,7] or by binning the TFBSs according to their position [8,9]. One weakness of these approaches is that they process the TFBSs in a binary way; either a site is present within the window, or it is not. Within the region defined by the window or bin itself the distribution of sites is ignored, and sites at the edge of the region contribute as much to the score as do sites located at the center. This causes such approaches to be highly dependent on window sizes, and – especially for smaller window sizes – susceptible to noise: moving the region or changing its size even just a few bases can drastically change the score. Alternative methods for combining positional information and nucleotide motifs, such as one based on positional word counting [10,11], are not suitable for application on smaller datasets where counts of TFBSs tend to be too low to process in such a way.

Here we introduce a novel method for the prediction of locally enriched TFBSs in sets of promoter sequences of co-regulated genes. Our approach is based on the Parzen window technique for density estimation, and is capable of converting predicted TFBSs in even small sets of promoters into continuous functions that reflect local TFBS enrichment. Usage of different window function widths allows for the detection of both TFBS motifs with loosely defined positional preferences as well as TFBSs that require very precise positioning. Furthermore, a random sampling technique is used to incorporate genome-wide tendencies into the evaluation of significance of local enrichment. Application on artificial and real datasets showed that our method compares favourably to other methods and measures. We also applied our method to a number of promoter sequence sets regulating tissue- or condition-specific expression profiles, and detected regions with local enrichment of TFBSs that could not be detected using a more standard TFBS over-representation analysis. We illustrate the validity of our findings and how our approach can be used for further dissection of the architecture of regulatory regions.

## Methods

Our proposed method for detection of locally enriched regulatory motifs is described below. A workflow of our approach as well as details not covered below can be found in the Supplementary Methods section and Figure S1 (Additional file 1).

### Parzen window-based scoring of local TFBS enrichment

We start with a set *S* of *N* promoter sequences, indexed by *s* = 1, 2, …, *N*. For each sequence *s* its *m*_*s*_ associated TFBSs are represented by their location relative to a landmark within each sequence, 

$L_{S}=\left\{l_{s,1},l_{s,2},\ldots ,l_{s,m_{s}}\right\}$. In this study the landmark is the TSS associated with each promoter sequence, and our goal is to find a region relative to the TSS within these promoter sequences that is significantly enriched in TFBSs.

We start the description of our approach by temporarily focusing on a window of size *h*, which is used to scan the set of sequences. The probability *P* that a TFBS will fall within a region *R* can be expressed as:

(1) $$P=\int_{R}p\left(x\right)\mathrm{dx}$$

The density function *p*(*x*) can be estimated empirically by

(2) $$p\left(x\right)=\frac{1}{n}\sum_{s=1}^{N}\sum_{i=1}^{m_{s}}\phi \left(x,l_{s,i}\;,h\right)$$

where *n* is the total number of TFBSs in the set of *N* sequences. Here *ϕ* is a kernel function referred to as the Parzen window function. In a simple sliding window approach *ϕ* could be defined as follows:

(3) $$\phi \left(x,l,h\right)=\{\begin{array}{l} \frac{1}{h}\;if\;\left|x-l\right|\leq h/2\\ 0\;otherwise \end{array}$$

In this case *p*(*x*) simply reflects the count of sites in the window around *x*.

In our approach, a first adjustment we made is to replace the simple Parzen window function of Eq. 3 with a smoother window function:

(4) $$\phi \left(x,l,h\right)=\frac{1}{\sqrt{2\pi {\left(h/2\right)}^{2}}}\exp\left(\frac{-{\left(l-x\right)}^{2}}{2{\left(h/2\right)}^{2}}\right)$$

That is, *ϕ* is a Gaussian function fit over each position *x* in the set of promoters, with the distance to predicted TFBSs determining their contributions to the score of *x*. The *h* value here is essentially a measure of the width of this Gaussian. Other window functions can easily be introduced.

Secondly, we adjusted the Parzen window approach so that *p*(*x*) is no longer an estimate of the density of TFBSs, but of the local enrichment of TFBSs in the region around position *x* in the promoter sequences. We define *p*_*s*_(*x*) to be the contribution of the TFBSs of sequence *s* to the local enrichment score at position *x*;

(5) $$p_{s}\left(x\right)=\sum_{i=1}^{\mathrm{ms}}\phi \left(x,l_{s,i},h\right)$$

and the total local enrichment score (*S*_*local*_) at position *x* over the entire set of sequences *S* is

(6) $$S_{\mathrm{local}}\left(x\right)=\sum_{s=1}^{N}\frac{1}{Z_{s}}p_{s}\left(x\right)$$

Here, *Z*_*S*_ is a normalization factor inspired by the Zero or One Occurrence Per Sequence (ZOOPS) model that is frequently used in motif detection programs [12]:

(7) $$Z_{s}=\max\left(1,\sum_{x=\mathrm{xstart}}^{\mathrm{xstop}}\mathrm{ps}\left(x\right)\right)$$

where *x*_*start*_ and *x*_*stop*_ indicate the start and stop coordinates of the region of interest relative to the TSS, respectively. In practice, if a sequence contains multiple predicted TFBSs, *Z*_*s*_ will be greater than 1, resulting in a reduced contribution per site in this sequence to the *S*_*local*_. This scaling factor was introduced to limit the effect of a single sequence containing high numbers of predicted TFBSs, especially in simple repeat sequences (see Additional file 1: Figure S2).

Note that the *S*_*local*_ is not a probability function and does not sum to unity. Using Equations 4 to 7, a local enrichment score can be calculated for each TFBS motif at each bp in the region from *x*_*start*_ to *x*_*stop*_. In practice we focused on the region from -2 kb to 1 kb around the TSS, but to avoid irregularities at the boundaries of this region we based calculations on TFBSs predicted in the region from -3 kb to +2 kb. Wider window functions lead to broad, slowly changing *S*_*local*_ values which are useful for detecting over-representation of TFBSs with relatively loosely defined regions of preference. Narrower window functions on the other hand result in sharper peaks in *S*_*local*_ values, allowing for the detection of local enrichment in narrow, precisely defined regions. In order to detect both broad and narrow regions of enrichment we used the values *h*/2 = 10, 20, 50, 100, and 200 bps.

### Evaluation of significance of local enrichment

The significance of observed *S*_*local*_ values was evaluated using a random sampling approach, in which we sample *N* sequences with their predicted binding sites from the genomic set of promoters. Sampling was done such that sampled sequences had a similar GC content composition as the input sequences, in an effort to limit biases (see below). Using Equations 4 to 7, for each position *x*, we calculated

$S_{\mathrm{local}}^{\mathrm{sampled}}$(*x*). We repeated this sampling a large number of times and for each position *x* we calculated two p-values: a position-dependent one, *P*_*dep*_(*x*), and a position-independent one, *P*_*ind*_(*x*). *P*_*dep*_(*x*) is defined as the proportion of sampled sets where 

$S_{\mathrm{local}}^{\mathrm{sampled}}$(*x*) ≥ *S*_*local*_(*x*), and *P*_*ind*_(*x*) is defined as the proportion of sampled sets where max _*x*'_(

$S_{\mathrm{local}}^{\mathrm{sampled}}$(*x*')) ≥ *S*_*local*_(*x*).

The use of the combination of both thresholds ensures that enriched regions are enriched in *S* in comparison to the same region in sampled sets (*P*_*dep*_), but also that the regions have a certain degree of enrichment irrespective of their position to the TSS (*P*_*ind*_). In order to reduce false positive predictions caused by multiple testing, we defined the thresholds for *P*_*dep*_(*x*) as a function of the window function width, with values of 0.001, 5e-4, 2e-4, 1e-4, and 5e-5 for scores calculated with *h*/2 = 200, 100, 50, 20, and 10 bps respectively. Here, more stringent thresholds for the smaller peak widths reflect the increasing number of independent tests performed as peak widths decrease. The *P*_*ind*_(*x*) threshold was set uniformly to 0.01. For every region within the sequences with start position *x*_*1*_ and end position *x*_*2*_ passing both *P* value restrictions we defined the corresponding region of local enrichment to be the region from *x*_1_ - *h*/2 to *x*_2_ + *h*/2.

### Removal of redundant locally enriched regions

Pairs of overlapping enriched regions detected for the same motif with the same window function width were merged together to form one single region. Redundancy between enriched regions detected using different window function width values was removed such that for each set of overlapping regions only 1 representative region was retained. For each region, the number of TFBSs contained was counted. Next, for each set of overlapping regions we retained the largest region, unless there were smaller regions containing more than 2/3 of the predicted TFBSs in the largest region. In the latter case we retained the smallest region containing more than 2/3 of the predicted TFBSs. This approach ensured that, where possible, narrower enriched regions containing high numbers of TFBSs were returned.

### A random sampling approach that takes into account GC content biases

At several points in this study we evaluate the significance of findings using a random sampling strategy. Observations made for the set of sequences *S* were compared to values obtained in a large number of sets of *N* sequences randomly sampled from the genomic set of sequences in a way that limits the difference in GC content between sampled sequences and the sequences in *S*. The p-value of observed values was estimated by the ratio of sampled sets having a higher value than the observed one.

First, we clustered the genomic set of promoter sequences by their GC content. Each sequence was represented by a vector of 20 values representing the GC content in bins of 100 bps in the region from -1 kb to +1 kb. Values for each bin were scaled to have mean 0 and standard deviation 1. Clustering was done using k-means clustering using the Hartigan-Wong algorithm, with *k* = 2 to 10. For each value of *k*, clustering was done 100 times using random initializations, each run had up to 100 iterations, and the result with the smallest sum of squares between samples and assigned centers was retained. The clusters obtained with *k* = 2 corresponded to sequences with high (especially proximal to the TSS) and low GC content, respectively. Clusters obtained by clustering with *k* > 2 corresponded to variations of both classes (see Additional file 1: Figure S3).

In our sampling approach, given a set *S* of *N* input sequences and a value of k*, we get the number of sequences in *S* belonging to each cluster, 

$\left\{c_{1},c_{2},\ldots ,c_{k^{*}}\right\}$, with

$$\sum_{i=1}^{k^{*}}$$

**Figure 1:**
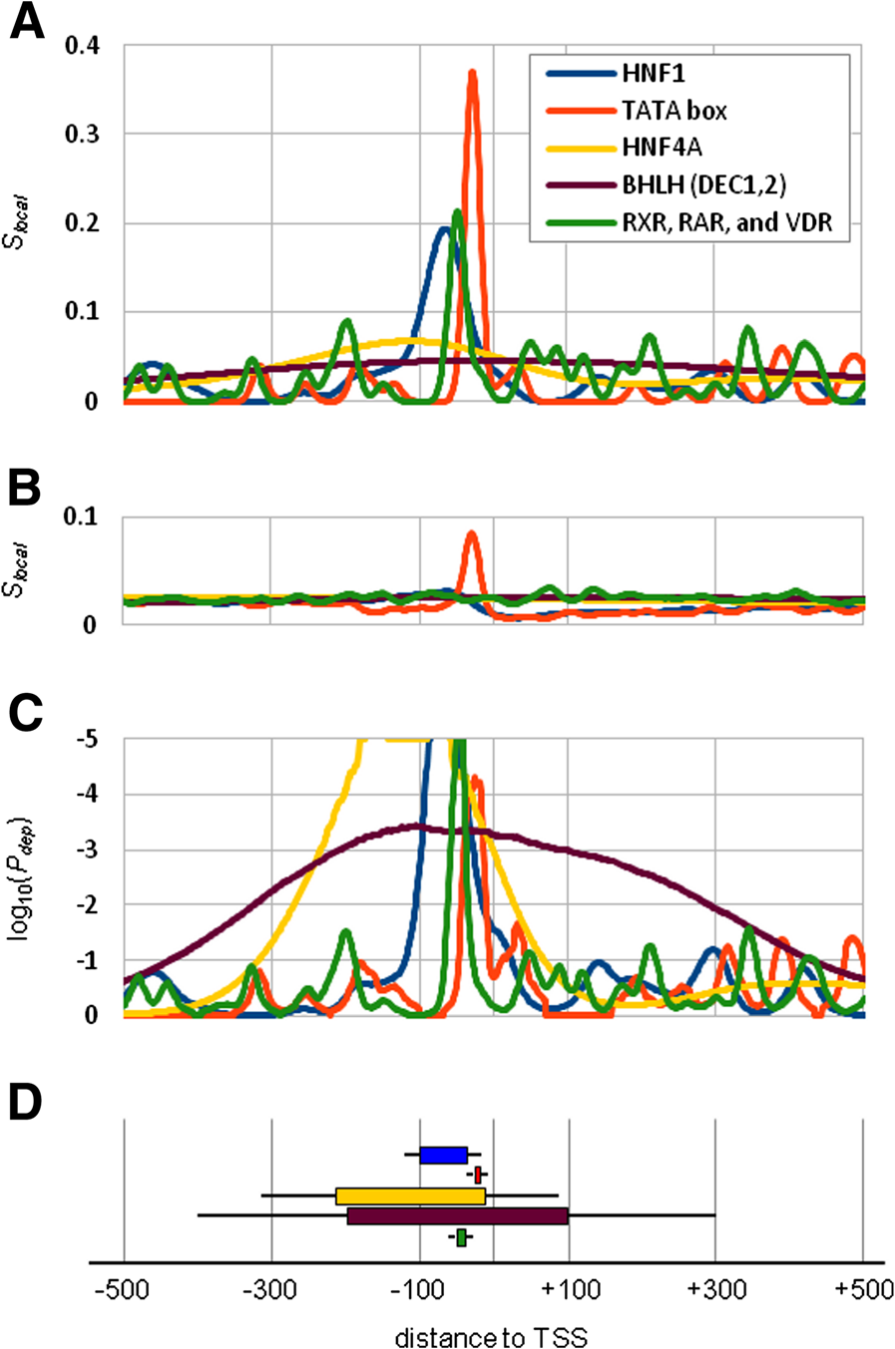
**The effect of the random sampling approach for minimizing GC content influences.** (**A**). The average GC content in bins of 100 bps in the region -1 kb to +1 kb is shown for 159 promoters of genes with small and large intestine-specific expression (black), and for the same number of sequences randomly sampled from the genomic set of promoters with *k* = 1 (blue), *k* = 2 (red), and *k* = 3 (green). Values of sampled sets are mean values with bars representing the standard deviation based on 500 sampled sets. (**B**). For the same dataset, the average RMSD of GC content is shown for *k* = 1 to 10. In this case k* is set to 2.

*c*_*i*_ = *N*, and randomly sample *c*_*i*_ sequences from each cluster of the genomic set of sequences. Here, k* represents the optimal *k* value, chosen for each set of input sequences *S* in a way that limited the difference in GC content profiles between *S* and sampled sequences. For determining k*, for each *k* value (*k* = 2 to 10), we randomly sampled *c*_*i*_ sequences from each cluster of the genomic set of sequences, and, for the thus obtained *N* sampled sequences, calculated the average GC content in each bin of 100 bps. The root-mean-square deviation (RMSD) between these GC content values and the average GC content values of the sequences in *S* is calculated. This sampling was repeated 1000 times, allowing us to calculate the average RMSD and its standard deviation (SD) (see Figure 1B for an illustration). The estimate of k* was then:

(8) $$k*=\arg\min_{k}\left\{k|\mathrm{RMS}D_{k}\leq \mathrm{RMS}D_{k+1}+SD_{k+1}\right\}$$

which is inspired by the Gap statistic proposed to estimate the optimal *k* in k-means clustering [13].

### Over-representation index

For the purpose of comparison, as an indication of global enrichment (as opposed to local enrichment) of a TFBS in *S* we employed the Over-Representation Index (*ORI*) [14], based on TFBSs predicted in the 1000 bps upstream of TSSs.

(9) $$\mathrm{ORI}=\frac{\mathrm{Densit}y_{S}}{\mathrm{Densit}y_{\mathrm{genomic}}}\times \frac{\mathrm{Proportio}n_{S}}{\mathrm{Proportio}n_{\mathrm{genomic}}}$$

where *Density*_*S*_ is the number of predicted TFBSs in *S* divided by *N*, *Density*_*genomic*_ the number of predicted TFBSs in the genomic set of promoter sequences divided by the total number of promoter sequences, and *Proportion*_*S*_ and *Proportion*_*genomic*_ the ratio of sequences that have at least 1 predicted TFBS, in *S* and the genome-wide set of promoters, respectively. The significance of observed *ORI* values is evaluated using a sampling approach equivalent to the one used for local enrichment scores, with *P*_*ORI*_ being the proportion of random samples with higher ORI scores than the value observed in *S*. See (Additional file 1: Figure S4) for a flowchart of this approach. After application on artificial datasets, and based on our experience in previous analyses, we set the threshold for *P*_*ORI*_ to 0.01.

### Comparison to existing methods and measures

We compared the performance of our approach to that of existing methods and measures for the prediction of local and global enrichment, using artificial as well as real data. A number of realistic, artificial sequence datasets were prepared in which instances of known regulatory motifs were inserted into specific regions with respect to the TSSs. Real datasets consisted of 6 sets of genes with similar expression profiles in dendritic cells upon stimulation with LPS, for which ChIP-seq time-course data is available for 25 TFs [15]. Using the ChIP-seq data we inferred the TFs controlling the expression of each set of genes. On these datasets we applied the methods and evaluated their performance in terms of precision, recall, and F-measure. For a more detailed description of the construction of the artificial and real datasets, the methods and measures we included in the comparison, and the measures of performance we used, we refer to the Supplementary Methods section (Additional file 1).

### Summary of additional materials and methods

For a more detailed description we refer to the Supplementary Methods section (see Additional file 1). In brief, sets of co-expressed genes were defined based on micro-array gene expression data for 79 human and 61 mouse tissues and cell types from the GNF GeneAtlas dataset [16], and for mouse dendritic cells (DCs) stimulated with a number of immune stimuli [17]. For all genes of the human (hg19) and mouse (mm9) genome, we scanned regions from -3 kb to +2 kb around the TSS for sites for a set of non-redundant, vertebrate position weight matrices (PWMs) constructed from TRANSFAC [18] and JASPAR [19] PWMs. Analysis of local enrichment of TFBSs was based on these predicted sites.

Evolutionary conservation of TFBSs within enriched regions was evaluated using PhastCons scores as available on the UCSC Genome Browser [20]. Z scores for PhastCons scores corresponding to bases included in TFBSs were calculated based on the average and standard deviation of PhastCons scores of an equal number of bases located at equal distances to randomly selected TSSs.

Enrichment of weak TFBSs in locally enriched regions was evaluated by comparing the count of weak sites within predicted regions in *S* with the count in randomly sampled sets of sequences.

## Results and discussion

### Parzen window-based detection of local enrichment of TFBSs

The starting point for many TFBS analyses is a set of predicted TFBS in a set of regulatory sequences believed to be under the control of the same regulatory mechanism. Finding local over-representation of TFBSs in a set of sequences can be thought of as finding a region at a certain distance from a landmark, such as the TSS, in which there is a significant enrichment or increase in density of TFBSs compared to a reference set of sequences.

Various general approaches for the estimation of densities have been described, such as frequency histograms and k_n_-nearest-neighbor estimates [21]. Here, we focused our attention on the Parzen window approach, a non-parametric technique where the density estimate is obtained by summing over all samples weighted by a function of choice, the so-called window function [22]. Parzen widow approaches have been used in bioinformatics for ChIP-seq peak calling [23], but to our best knowledge it has never been used for the analysis of regulatory motifs. In our analysis, samples consist of predicted TFBSs in promoter sequences of co-expressed genes, and as a window function we use a Gaussian function of the distance to each TFBS. We choose a Gaussian function because it results in a smooth enrichment score, and because it has only one additional parameter to specify: the width of the Gaussian. We made a number of adjustments to the original Parzen window approach (see Methods section), which allow us to estimate local enrichment scores (*S*_*local*_) for each TFBS. A sampling approach is then used to estimate the significance of observed scores in a way that takes into account GC content profiles of sequences.

**Figure 2:**
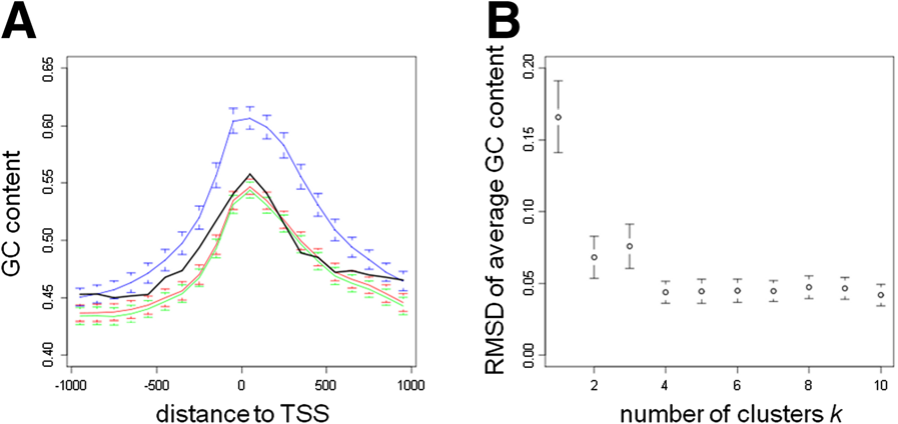
**Illustration of our approach for prediction of local TFBS enrichment.** (**A**). For 159 mouse genes with high expression in small and large intestine we show the local enrichment scores in the region -500 to +500 around the TSS for 5 TFBS motifs. The *h*/2 values were 10 bps for TATA and RXR/RAR/VDR; 20 bps for HNF1; 50 bps for HNF4; and 200 bps for bHLH. (**B**) The expected local enrichment scores for the same motifs based on the genome-wide set of promoter sequences. *h*/2 values are as in (**A**). (**C**) *log*_10_(*P*_*dep*_) values for the local enrichment scores for the same 5 motifs in the same set of promoters. (**D**). Visual representation of the locally enriched regions for the 5 motifs. Boxes represent bases with significant scores and lines at both sides represent the *h*/2 values used. Enriched regions correspond to the regions covered by the boxes and lines.

To illustrate our approach we first focus our attention on the predicted sites for a selection of 5 PWMs in the promoters of 159 mouse genes with tissue-specific expression in small and large intestine. A first important point is that for each of these 5 motifs, a region of local enrichment was found (Figure 2D), while only one of them (HNF4) was found to be significantly over-represented by a standard over-representation approach. Secondly, between the 5 motifs there is a diversity in the width of the region of enrichment (Figure 2A): the TATA box and RXR/RAR/VDR motifs show very narrow *S*_*local*_ peaks, while the curves of the HNF4 and especially the bHLH motifs (DEC1,2) show very broad peaks. For comparison, the curves for expected *S*_*local*_ values based on the genome-wide set of promoter sequences are shown in Figure 2B. The peak for the TATA box corresponds to its known location around positions -30 to -25. Other motifs show a smaller degree of variation in function of the position relative to the TSS, and some have a more or less uniform distribution of sites. In order to take into account such genome-wide tendencies, we employ a sampling approach for the evaluation of significant local enrichment (see Methods section). Figure 2C shows the position dependent p-values (*P*_*dep*_) estimated by this sampling approach. Importantly, the fact that the TATA box is found to be significantly enriched in the promoters of intestine-specific genes implies that this motif is significantly enriched in the region just upstream of the TSS, even taking into account its strong genome-wide preference for this region (Figure 2B), suggesting that the TATA box plays a role in the regulation of these genes. In this way, our approach is able to find regulatory motifs that are missed by standard approaches, it can detect both broad and narrow regions of local enrichment of motifs, and it takes into account genome-wide tendencies during the evaluation of significance of enrichment.

Several studies have indicated differences in GC content and CpG scores between housekeeping genes and tissue- or condition-specific genes [24,25], and a recent study has suggested that analysis of regulatory regions can lead to better results when treating CpG island-associated sequences and non-CpG island-associated sequences separately [26]. Since PWMs with high (low) GC content naturally tend to have more hits in sequences with high (low) GC content, local regions of higher (lower) GC content in an input set of sequences can easily result in apparent local enrichment of GC-rich (GC-poor) TFBSs. To avoid such biases we designed a way to evaluate the significance of peaks using a sampling approach in which sampled sequences have a GC content profile similar to that of the input sequences (see Methods section). Figure 1 illustrates the importance of this approach using the set of mouse promoters with high activity in small and large intestine as an example. Figure 1A shows the GC content profile in the intestine-specific promoters, as well as in sequences randomly sampled using *k* = 1, 2, 3 cluster(s). If we were to ignore GC content profiles of sequences, sampling from the genomic set of sequences (equivalent to *k* = 1) would result in sampled sets having a significantly higher GC content than the input sequences, especially around the TSS. This in turn would lead to high numbers of false positive enriched regions for AT-rich TFBSs. However, when clustering genomic sequences into 2 clusters according to their GC content profile (*k* = 2), and sampling according to the number of input sequences in each cluster, the difference in average GC content between sampled and input sequences is strongly reduced. Doing the same using 3 clusters (*k* = 3) does not further decrease the difference in this particular case. Figure 1B shows the discrepancy between GC content of the same set of intestine-specific promoters and sampled sets of promoters as a function of the number of clusters *k* used to sample. Although the RMSD has a tendency to decrease as *k* increases, higher *k* values also result in smaller sets of promoter sequences to sample from. As a compromise, we therefore used the clusters obtained using k*, the smallest *k* value where the RMSD is smaller than the RMSD + SD of *k* + 1 clusters. In the case of promoters with high expression in small and large intestine, the random sampling for the evaluation of significance of local enrichment of TFBSs was thus done according to k* = 2. Since the existence of high-CG genes and low-CG genes is well established, k* = 1 was not considered. The k* values were 2 in 36 out of 44 sets of co-expressed genes in mouse, and in 17 out of 32 sets in human, indicating that in many cases a simple distinction between GC-rich and GC-poor sequences is sufficient. For a number of sets, however, high k* values were found (Additional file 1: Table S1, Additional file 1: Table S2 and Additional file 1: Table S3).

### Comparison to existing methods and measures

We compared the accuracy of our method to that of other methods and measures for the prediction of local and global motif enrichment, using artificial sequence datasets and real datasets based on RNA-seq and ChIP-seq data [15]. Both artificial and real datasets were constructed in a way that reflects a typical application of our method, e.g. promoters of sets of genes thought to be under the control of similar regulatory mechanisms (see Methods, and the Supplementary Methods section and Additional file 1: Tables S4). An overview of the precision, recall, and F-measure of each method and measure is shown in Table 1. The comparison shows that our method compares favourably to other methods. Recall is relatively high (0.755 and 0.371), while precision is the highest of the investigated methods for both artificial and real datasets (0.609 and 0.757 respectively). Interestingly, our method also had relatively high performance when using a simple uniform window function, though both recall and precision were lower than when using Gaussian-based window function. Although a number of approaches had higher recall values than our method, these methods tend to also have considerably lower precision. When methods for the prediction of global enrichment are run with more stringent thresholds in order to improve precision, recall drops and especially artificial TFBSs inserted with lower insertion rates (10% and 20%) tend to be missed (data not shown). Other methods for the prediction of positional preferences or local enrichment of TFBSs suffer in general from low precision, although we have to point out that several methods and measures were originally designed for slightly different purposes than the one investigated here. The prediction of *de novo* motifs is in general regarded as a much harder problem than the detection of enrichment of known motifs. This is reflected in the low recall and low precision of A-GLAM and FREE [5,27], two methods which do not use an input set of PWMs, but are based on the analysis of k-mers.

**Table 1:** Overview of performance of several methods and measures for prediction of motif enrichment on artificial and real datasets

**Method or measure**	**Type**	**Artificial data**			**Real data**			**Reference**
		**Recall**	**Precision**	**F-measure**	**Recall**	**Precision**	**F-measure**	
LocaMo Finder (Gaussian)	local	0.755	0.609	0.674	0.371	0.757	0.498	this study
LocaMo Finder (uniform)	local	0.727	0.519	0.606	0.343	0.723	0.465	this study
RSAT (Binomial distribution) (^$^)	global	0.714	0.285	0.408	0.429	0.440	0.434	RSAT [40]
ORI (**)	global	0.677	0.386	0.492	0.343	0.563	0.426	this study
Hypergeometric distribution (*)	global	0.745	0.272	0.399	0.400	0.450	0.424	AlignACE [41]
Fisher’s exact test (*)	global	0.747	0.276	0.403	0.400	0.443	0.420	oPOSSUM [42]
ORI (*)	global	0.768	0.258	0.387	0.429	0.407	0.417	this study
RSAT (Binomial distribution) (^$$^)	global	0.591	0.498	0.541	0.271	0.607	0.375	RSAT [40]
Hypergeometric distribution (***)	global	0.605	0.522	0.560	0.243	0.706	0.361	AlignACE [41]
Fisher’s exact test (***)	global	0.605	0.530	0.565	0.243	0.667	0.356	oPOSSUM [42]
Casimiro *et al*.	local	0.727	0.053	0.099	0.629	0.132	0.218	[9]
Berendzen *et al*.	local	0.859	0.044	0.083	0.786	0.093	0.167	[1]
Vardhanabhuti *et al*.	local	0.409	0.079	0.133	0.314	0.090	0.139	[3]
FIRE (Information content)	global	0.586	0.342	0.432	0.100	0.200	0.133	FIRE [43]
TFM-Explorer	local	0.432	0.145	0.217	0.186	0.076	0.108	[6]
FREE	local	0.155	0.182	0.167	0.029	0.013	0.018	[5]
A-GLAM	local	0.032	0.259	0.057	0.000	0.000	NA	[4,27]

For each method or measure the type of measure (“local”: local enrichment of positioning; “global”: global enrichment), the recall, precision, and F-measure is given for the artificial and real datasets, as well as a reference. Methods are sorted by decreasing F-measure obtained on the real datasets. (*) P value threshold 0.01; (**) P value threshold 0.001; (***) P value threshold 1e-4; (^$^) sig threshold 0; (^$$^) sig threshold 2.

In addition, we observed how our method significantly increases the accuracy of PWM-based TFBS predictions. When we assume that only predicted TFBS present inside bound regions of the TF in question (as defined by ChIP-seq data) are truly functional, and regard any other predicted TFBSs as false positives, PWM-based TFBS predictions resulted in a recall of 33.1%, with a low precision of 5.0%. However, when we filtered the PWM-based predicted TFBSs using the predicted regions of enrichment for the regulatory motif in question, while recall dropped 3.7-fold to 8.9%, there was a more than 7-fold increase in precision to 37.4%, resulting from a strongly reduced number of false positive predictions.

### Application on a large number of gene sets

Next, we applied our approach on a large number of sets of genes with tissue- and cell type-specific expression, in human and mouse [16]. Genes were clustered into 44 and 32 clusters of co-expressed genes for the mouse and human case, respectively. Promoter regions for all genes were scanned using a set of 198 non-redundant vertebrate PWMs, and we predicted local TFBS motif enrichment in each of the clusters using our approach, as well as TFBS enrichment using a standard motif over-representation approach.

In total our approach predicted 269 and 190 regions with local enrichment of a regulatory motif in the mouse and human GNF GeneAtlas datasets, respectively (see Additional file 2: Tables S5 and Additional file 2: Table S6). The fact that our approach could find locally enriched motifs that could also be detected using standard approaches demonstrates the robustness of our approach (see Additional file 1: Table S7). For example, sites for two ETS family TFs, including PU.1, and for NF-κB, and IRFs were found in various immune-related cell types [28-30], and sites for HNF1 and HNF4 in liver-specific and liver/kidney-specific genes [31,32]. Moreover, Table 2 shows a small selection of locally enriched motifs that could not be detected by the standard motif over-representation approach for the mouse clusters (see Additional file 1: Tables S8 and Additional file 1: Table S9 for more results). For example, we found enrichment of binding sites of ETS family TFs in promoters driving high expression in mouse B cells and T cells, and a peak for a CREB binding motif in a set of promoters associated with genes with preferential expression in mouse testis. Note that in many cases, motifs with local enrichment also show a certain degree of general enrichment (*P*_*ORI*_ values roughly in the range 0.01 to 0.20), but on a level that cannot be regarded as significant (*P*_*ORI*_ threshold = 0.01). These results clearly indicate that our approach is able to detect regulatory features that are missed by a standard motif over-representation analysis, and that in many cases the detected regulatory motifs are known to play a role in the dataset in which they were detected.

**Table 2:** **A selection of regions of local enrichment that could not be found using standard TFBS over**-**representation analysis**

**Tissues or cell types (cluster index)**	**Transcription factor**	**Region of enrichment:**	**P**_**ORI**_	**References**
		**x**_**1 **_**to x**_**2 **_**( *h/ *2)**		
B cells, T cells (2)	ETS domain TFs, including SPI1 or PU.1	-10 to 0 (10)	0.15	[44,45]
B cells, T cells (2)	HIF1	55 to 200 (200)	0.11	[46]
testis (10)	RFX1	-91 to 129 (200)	0.012	[47,48]
testis (10)	CREB-binding TFs, including ATF family	-148 to 31 (100)	0.035	[49,50]
liver (16)	Cux1 (CR3 + HD)	-103 to -90 (10)	0.037	[51]
small and large intestine (19)	HNF1	-93 to -37 (20)	0.012	[52]
small and large intestine (19)	RXR, RAR, and VDR	-52 to -43 (10)	0.065	[53,54]
testis (22)	MYB family TFs	-72 to 95 (100)	0.025	[55,56]
testis (22)	heat shock factors	-58 to 217 (200)	0.032	[57]
skeletal muscle (42)	THR alpha and beta	-30 to -15 (50)	0.025	[58,59]

A selection of regulatory motifs is shown for which regions of local enrichment were detected in mouse tissues and cell types of the GNF GeneAtlas dataset. The tissue, the start and stop position of the region, the *h*/2 used, the ORI p-value, and references supporting the role of the regulatory motif in the tissue in question are shown.

### General tendencies of local TFBS enrichment

**Figure 3:**
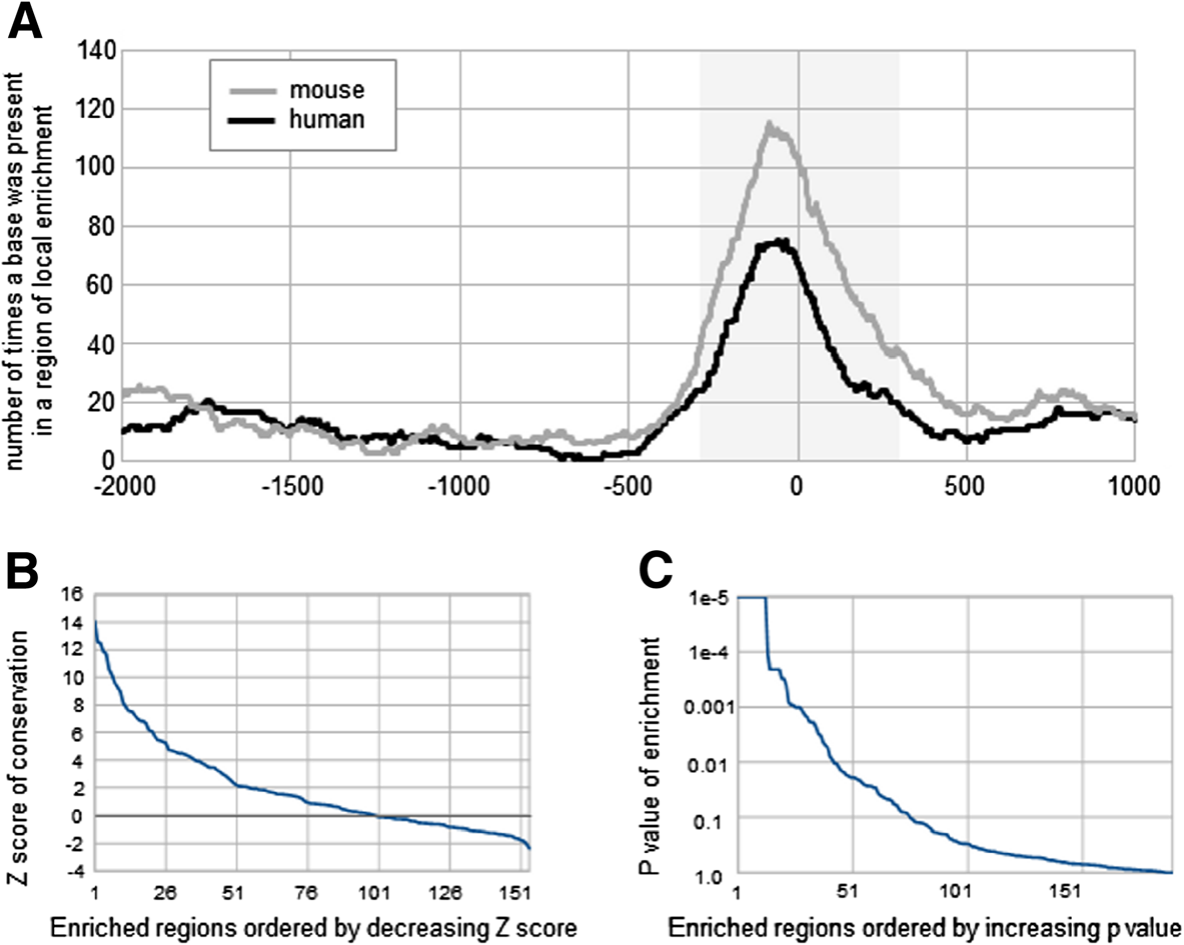
**General trends of significantly locally enriched regions detected in the GNF GeneAtlas gene sets.** (**A**) For each base in the region from -2 kb to +1 kb, the number of times it was found to be included in regions of local enrichment is shown, for 32 human and 44 mouse gene sets. The grey region indicates the region from position -300 to +300 where local enrichment was often found. (**B**) Human enriched regions sorted by Z score of PhastCons scores of the TFBSs within each region. (**C**) Human enriched regions sorted by p-value of enrichment of weak TFBSs within each region.

In general, locally enriched regions were found to be present proximal to the TSS, roughly in the region -300 to +300 with a peak around position -100 (Figure 3A). Interestingly, in the human case we observed a region that is relatively poor in enriched regions, roughly between positions -700 to -500, suggesting an upper limit for distances over which positioning of TFBS relative to the TSS is of biological importance. In mouse too, the number of times a nucleotide was included in an enriched region dropped to a low basal level for positions upstream of position -500. A similar limit was observed for the region downstream of the TSS, although both in human and in mouse the slope of the curve is not as steep. While the peak in the region just upstream of the TSS seems highly significant and suggests a recommended region for regulatory motif analysis roughly between positions -500 and +500, it is possible that it is partly caused by a bias of past studies to focus mainly on this region for the identification of TFBSs. This might in turn bias the PWM data we used for detection of local enrichment of TFBSs. On the other hand, given the flexibility of the DNA double helix it is unlikely that very precise positioning of TFBSs is necessary at larger distances from the TSS.

### Regulatory motifs show a preference for similar regions across different sets of promoters

An additional indication of the biological validity of our findings is the fact that for many motifs we found local enrichment in similar regions in different clusters of genes. One example is the TATA box, which is known to be present at a specific distance from the TSS and which we found to be significantly enriched in the region around positions -30 to -25 in two sets of promoters. We also observed similar tendencies for motifs that are not generally known to be under strong positional restrictions. Some examples are ETS family TFBSs, which we detected concentrated around the region immediately upstream of the TSS in 6 human and 9 mouse clusters (Table 3, and Additional file 1: Table S10), and NRF-1 binding sites, which we found in 4 human and 5 mouse clusters, here too in all cases in the region immediately upstream of the TSS. Importantly, not all motifs were enriched in the upstream regions: Zfx motifs, for example, were found to be enriched in regions roughly between 50 to 400 bases downstream of the TSS in 4 clusters. Standard motif over-representation analyses are likely to miss such regulatory patterns, as this region is usually not included in the input.

**Table 3:** Regions with local enrichment of TFBSs of ETS domain TFs

**Species**	**Tissues or cell types (cluster index)**	**Region of enrichment:**
		**x**_**1 **_**to x**_**2 **_**( *h */2)**
human	T cells, NK cells (5)	-141 to 51 (100)
human	721 B-lymphoblasts, BM CD34+ cells (11)	-174 to 42 (100)
human	721 B-lymphoblasts, BM CD34+ cells (12)	-151 to 37 (100)
human	B cells, Burkitt's lymphoma (13)	-148 to 23 (100)
human	BM CD34+ cells, 721 B-lymphoblasts (14)	-81 to 10 (50)
human	NK cells, T cells (15)	-97 to 34 (50)
mouse	B cells, T cells (2)	-106 to 17 (50)
mouse	skeletal muscle, heart (4)	-161 to 26 (100)
mouse	thymus, ovary (6)	-137 to 18 (100)
mouse	testis (10)	-133 to -58 (100)
mouse	T cells, B cells (12)	-133 to -98 (100)
mouse	oocyte, fertilized egg (25)	-139 to 42 (100)
mouse	oocyte, fertilized egg (34)	-240 to 34 (200)
mouse	testis (35)	-178 to 71 (200)
mouse	oocyte, fertilized egg (37)	-77 to -11 (50)

Region with local enrichment of ETS domain TF binding motifs were found in different sets of sequences. The species, and the tissues and cell types associated with the promoters in which the motif was found are listed, as well as the regions of local enrichment.

### TFBSs in locally enriched regions tend to have high evolutionary conservation

**Figure 4:**
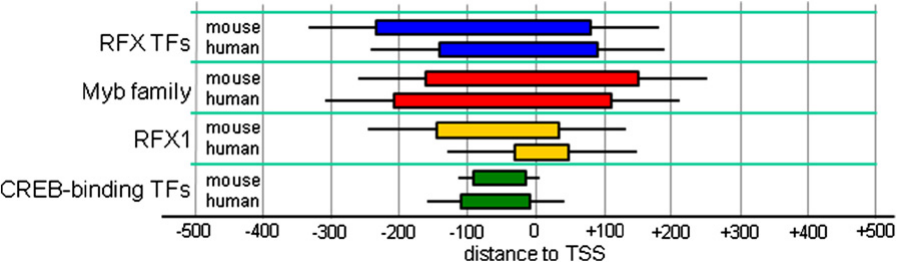
**Similar regions of local enrichment were detected in human and mouse promoters.** For 4 regulatory motifs, enriched regions predicted in mouse and in human genes with testis-specific expression are shown.

Another illustration of the validity of our findings is the relatively high evolutionary conservation of TFBSs present within enriched regions, compared to nucleotides within predicted TFBSs at the same position relative to TSSs of randomly sampled genes (Figure 3B; see also Supplementary Methods section in Additional file 1). Among the 154 enriched regions containing ≥ 10 TFBSs that were detected in the human GNF GeneAtlas sets, 74 (48.0%; expected: 24.5; p-value: 1.1e-20) contain TFBSs that are on average more conserved (Z score ≥ 1) than randomly picked positions, while only 22 (14.3%) contain sites that are on average less conserved (Z score ≤ -1). Similarly, for mouse datasets, we found 92 out of 205 (44.9%; expected: 32.6; p-value: 1.2e-22) regions with highly conserved sites, and only 23 (11.2%) with low conservation (Additional file 1: Figure S5A). In both human and mouse, the vast majority of the peaks with high conservation were located proximal to the TSS (data not shown). Among the relatively more conserved sites are the predicted sites for YY1 in genes specifically expressed in mouse thymus. The bases corresponding to the 80 YY1 sites predicted in the region -161 to +222 have an average PhastCons score of 0.69 (expected: 0.38, SD: 0.04, Z-score: 8.1). Evolutionary conservation was not only found on the level of predicted TFBSs, but also on the level of predicted regions of local enrichment, as illustrated in Figure 4 for promoters of testis-specific genes. For the sites of PWMs representing RFX1, RFX TFs in general, a CREB motif, and Myb family TFs, similar regions of enrichment were predicted in human and mouse sequences.

### Locally enriched regions tend to be enriched for weak TFBSs

Recently a number of studies, including computational studies using thermodynamic models, have illustrated the importance of weak TFBSs in the regulation of transcription [33-35]. Although PWM scores are believed to be correlated with the TF-DNA binding energy [36], the sheer number of sites having only moderately high PWM scores prohibits most computational methods from analyzing them in more detail. One advantage our approach offers is that we can restrict the region of interest to the region preferred by the stronger TFBSs, and evaluate if weaker binding sites show a similar local enrichment (see Supplementary Methods section in Additional file 1). Our analysis of weak TFBSs in the enriched regions found in the human clusters shows that 42 out of 190 regions (22.1%) show a significant enrichment (defined as p-value < 0.01) of weaker TFBSs (Figure 3C). Similarly, in mouse datasets, 49 out of 269 regions (18.2%) were enriched for weak TFBSs (Additional file 1: Figure S5). This result does not include the presence of stronger TFBSs, nor weaker sites overlapping with them. For example, promoters of genes expressed in human liver are enriched for HNF1 TFBSs in the region -225 to +49. Our analysis indicates that in addition to the 56 strong HNF1 sites, there are 49 weak TFBSs predicted in the region of local enrichment in the promoters in this set of genes, (expected: 19.8 sites, p-value < 1e-4).

### Application on sets of genes with differential expression upon TLR stimulation

The regulatory pathways controlling Toll-like receptor (TLR) signalling have been relatively well studied, and main regulators such as NF-κB and IRFs have been identified. However, it is likely that promoters of genes that are induced or repressed after TLR stimulation contain additional regulatory motifs that are still to be identified. From gene expression data taken from mouse DCs stimulated with 5 TLR ligands [17], we prepared 18 clusters of co-expressed genes. Applying our method to these clusters, we could detect locally enriched regions for the sites of CREB-binding TFs, NF-κB, and IRFs in clusters of transiently induced genes with peak expression around 2, 4, and 6 hrs after stimulation, respectively (See Additional file 2: Table S11). These motifs could also be found by standard motif over-representation analysis. However, our approach also detected a local enrichment for STAT binding sites in a cluster with induced expression peaking around 6 hours after stimulation [37], which could not be detected by the standard approach. In addition, we could make a number of findings that might offer hypotheses for further wet-lab experiments. One is the enrichment of TATA box motifs only in the set of promoters corresponding to genes with the fastest transient activation (peak induction at 2 hrs), which suggests that the TATA box plays a role in the rapid induction of these genes. Finally, a number of enriched regions downstream of the TSS were predicted. These include regions enriched in sites for Hmx3 (transiently induced, 2 hrs), RFX1 sites (transiently induced, 8 hrs), Mtf1 (late induction, 24 hrs), Zfp161 and E-box motifs (transiently repressed, 6–8 hrs).

## Conclusions

Although various studies have illustrated that in eukaryotic genomes *cis*-regulatory motifs can be positioned several kilobases or even megabases away from their target genes, it has also been reported that in a number of cases TFBSs show a tendency to be present at a more or less fixed distance with regard to the TSS. Nevertheless, in general, no clear positional preferences have been described for most regulatory motifs, even though such information could be extremely useful for their prediction.

Here, we present a novel method for detecting locally enriched TFBSs in the regulatory regions of sets of co-regulated genes. Our approach is based on a non-parametric approach for sample density estimation, with adjustments which allow it to detect sequence regions that have a significant local enrichment in TFBSs, on a 1 bp resolution. An online tool of our approach, which we call LocaMo Finder (Local Motif Finder), is available at http://sysimm.ifrec.osaka-u.ac.jp/tfbs/locamo/. Our approach evaluates both positioning and enrichment of TFBSs simultaneously, using a set of control sequences as a reference. We implemented our method for the estimation of significance of enrichment in a way that takes into account GC content profiles of the input set of promoter sequences. Evaluation of significance is done against randomly selected promoters that have GC content profiles similar to the input sequences. We showed how our approach has a high accuracy compared to other methods and measures for local and global motif enrichment.

We applied our method on a large number of sets of genes with tissue- or cell type-specific expression, as well as on a number of sets of genes with similar expression profiles after TLR ligand stimulation in mouse DCs. Detected regions of local enrichment of TFBSs are supported by known regulatory interactions reported in literature, as well as by the observation that several regulatory motifs are found to be locally enriched in similar regions in different sets of promoters. In addition, detected regions tend to contain TFBSs with higher evolutionary conservation than expected, and they also tend to be enriched for weak TFBSs. Together, these results illustrated the usefulness and validity of our approach.

Approaches for finding local enrichment of TFBSs heavily rely on TSS annotations. Recent studies have shown that a large fraction of genes have several TSSs, and that different TSSs might allow different amounts of variation in the bases from which transcription is initiated [38,39]. Future approaches that incorporate such features are likely to present more biological insights into the relationship between TFBS positioning and transcription initiation and TSS variability.

## Abbreviations

DC: Dendritic cell; ORI: Over-representation index; PWM: Position weight matrix; RMSD: Root-mean-square deviation; SD: Standard deviation; TF: Transcription factor; TFBS: Transcription factor binding site; TLR: Toll-like receptor; TSS: Transcription start site; ZOOPS: Zero or one occurrence per sequence

## Competing interests

The authors declare that they have no competing interests.

## Authors’ contributions

AV conceived of the study and performed the bioinformatics analysis, and prepared the manuscript. YK, ST, and KMA contributed to statistical analysis, discussion and interpretation of results and with drafting the manuscript. SA and DMS supervised the project and helped with discussion and interpretation of results and with drafting the manuscript. All authors read and approved the final manuscript.

## Supplementary Material

Additional file 1 **Supplementary text and figures.** A file containing supplementary material, including detailed description of methods and results, as well as supplementary figures and tables. Click here for file

Additional file 2 **Supplementary Tables S5, S6, and S11.** A spreadsheet file containing predicted regions of local enrichment and globally enriched TFBS motifs for human and mouse GNF GeneAtlas datasets, and for the sets of promoters obtained from TLR-stimulated mouse DC cells. Click here for file
