# Supplementary material for: A Parzen window-based approach for the detection of locally enriched transcription factor binding sites
Source: BMC Bioinformatics. 2013 Jan 21;14:26. doi: 10.1186/1471-2105-14-26 (PMC3602658; doi:10.1186/1471-2105-14-26)
Supplement: Additional file 1 — Supplementary text and figures. A file containing supplementary material, including detailed description of methods and results, as well as supplementary figures and tables. [file 1471-2105-14-26-S1.doc]

Supplementary material for “A Parzen window-based approach for the detection of locally enriched transcription factor binding sites”

Contents

[Supplementary methods 1](#__RefHeading___Toc344639167)

[Co-expressed sets of genes 1](#__RefHeading___Toc344639168)

[Promoter sequences 2](#__RefHeading___Toc344639169)

[Prediction of transcription factor binding sites 2](#__RefHeading___Toc344639170)

[Comparison to existing methods and measures 3](#__RefHeading___Toc344639171)

[Construction of realistic, artificial sequence datasets 3](#__RefHeading___Toc344639172)

[Construction of datasets based on real experimental data 3](#__RefHeading___Toc344639173)

[Overview of methods and measures included in comparison 4](#__RefHeading___Toc344639174)

[Measures of performance 6](#__RefHeading___Toc344639175)

[Enrichment of weak binding sites 8](#__RefHeading___Toc344639176)

[Evolutionary conservation of predicted binding sites within locally enriched regions 8](#__RefHeading___Toc344639177)

[Supplementary figures and tables 10](#__RefHeading___Toc344639178)

[References 28](#__RefHeading___Toc344639179)

# Supplementary methods

Details of our analysis not covered in the main paper can be found below. For a description of the central part of the analysis we refer to the Methods section of the main paper. A visual representation of the workflow of our approach can be found in Supplementary Fig. S1. The workflow of the standard transcription factor binding site (TFBS) analysis, which is not able to predict local enrichment of TFBSs and which we used for comparison, is shown in Supplementary Fig. S4.

## Co-expressed sets of genes

We obtained micro-array gene expression data for 79 human and 61 mouse tissues and cell lines from the GNF GeneAtlas dataset [1], and for mouse dendritic cells (DCs) stimulated with a number of immune stimuli [2]. For each dataset raw intensity data were processed to calculate robust multi-array average (RMA) values. Genes with at least 3-fold differential expression were selected. Expression values for each gene were rescaled to mean 0 and standard deviation 1, and dimension reduction was performed with principal component analysis. Gene expression patterns were hierarchically clustered with Ward’s algorithm and divided into subclusters. All analyses were performed using R and Bioconductor. See Supplementary Tables S1, S2 and S3 for a simple annotation of the obtained clusters of co-expressed genes.

For the mouse GNF GeneAtlas data, we obtained 8,443 genes in 44 clusters (see Supplementary Table S1). For the human GNF GeneAtlas data, we obtained 10,415 genes in 32 clusters (see Supplementary Table S2). Finally, for the mouse DCs dataset, we obtained 1,675 genes in 18 clusters (see Supplementary Table S3).

## Promoter sequences

We used a combination of DBTSS [3], CAGE data [4], and annotation data from the UCSC Genome Browser [5] to define TSS positions for both human and mouse genes, as described before [6]. We extracted the regions from -3kb to +2kb around the TSS of each gene of the hg19 and mm9 version of the human and mouse genome, respectively. In order to avoid biases in results caused by highly similar pairs of sequences, we iteratively removed a sequence if it was similar to one or more other sequences within the set S. In practice we ran an all-to-all BLAST alignment for all sequences, and used BLAST E values as indicator for sequence similarity, with a threshold value of 1e-10.

## Prediction of transcription factor binding sites

From the TRANSFAC [7] and JASPAR [8] databases all vertebrate position weight matrices (PWMs) were extracted. Similarities between PWMs were evaluated using tomtom [9], and redundancies were removed in a step-by-step fashion: for each pair of similar PWMs (tomtom E-value < 1 and at least 75% of the positions in the 2 PWMs overlapping) the PWM with lower information content was removed. This resulted in a set of 198 non-redundant PWMs. To each remaining PWM a label was assigned reflecting the transcription factors (TFs) or motifs it represents.

For each PWM a score threshold was set in a way that results in about 1 hit per 5000 bps in the genomic set of promoter sequences, and the genomic set of sequences was scanned for TFBSs. Locations of predicted TFBSs were defined as the center of the oligonucleotides with a PWM score higher than the corresponding PWM threshold. At positions where 2 or more overlapping sites for the same PWM were found, sites with lower PWM scores were removed.

## Comparison to existing methods and measures

### Construction of realistic, artificial sequence datasets

In order to compare the performance of our method with that of other methods and measures for the prediction of local and global enrichment, we prepared a number of artificial datasets, in such a way that they were as realistic as possible.

First, we randomized the genomic set of mouse promoter sequences by shuffling the sequence IDs within each class of sequences obtained by the k-means clustering based on GC content, using k = 10. Thus, sequences were assigned random IDs of other sequences with a similar GC content profile.

Next, from these randomized sequences we picked up the sequences corresponding to the actual 44 sets of co-expressed genes we obtained from the GNF GeneAtlas mouse data (see section “Co-expressed sets of genes” in the Supplementary methods). This gave us 44 artificial sets of sequences of the same size and similar GC content profiles to the actual sequence sets.

Finally, within each set of sequences, we inserted instances (TFBSs) of five randomly selected PWMs. TFBSs were inserted according to a normal distribution centered at a randomly decided position within the region -100 to +100, using a variety of standard deviations (from 5 bps to 500 bps), with an insertion rate of 10%, 20%, 30%, 40%, or 50% of the sequences, respectively. Inserted motifs were generated using the probabilities specified by each PWM. In each sequence at most 1 instance of a PWM was inserted.

### Construction of datasets based on real experimental data

In addition to artificial datasets, we prepared a number of datasets based on experimental data taken from mouse dendritic cells before and after stimulation with LPS [10]. This data consists of gene expression data (RNA-seq based RPKM values at 0, 1, 2, 4, and 6h), as well as TF-DNA binding data for 25 TFs (ChIP-seq data-derived peaks at 0, 0.5, 1, and 2h). The 25 TFs include well known key regulators of the immune response as well as other TFs with high expression in DCs. RPKM values and predicted ChIP-seq peaks were used as provided in the original paper (GEO accession number GSE36104).

Data was processed to a typical input for our method, e.g. promoters of sets of genes thought to be under the control of similar regulatory mechanisms. We clustered 1283 genes with at least a 2-fold induction after stimulation, and with at least 1 time point with RPKM > 1, using hierarchical clustering with distances based on correlation of log(RPKM) values. Clustered genes were divided into 6 clusters (see Supplementary Table S4).

We collected the ChIP-seq derived binding regions of the 25 TFs over the 4 time points in the region surrounding the TSSs of Refseq genes (region -1kb to +1kb). We used the same threshold scores for significant binding levels as used in the original paper (26.9).

For each of the 6 clusters of genes, we compared the observed number of genes bound by each TF at each time point in the region -1kb to +1kb to the expected number based on the fraction of genes in the genomic set of genes bound by the same TF. We calculated a p value for enrichment of binding based on a binomial distribution, and defined a set of genes to be under the regulation of a TF if at any of the 4 time points the p-value based on the binomial distribution was smaller than 1e-4. Several associations fit with known key regulatory interactions, such as the central role of NF-κB, and strong enrichment of IRF family TFs in genes with late induction [10]. Finally, from our set of PWMs, we associated relevant PWMs with each of the 25 TFs, resulting in 6 sets of genes with expected regulatory motifs responsible for their transcriptional regulation.

### Overview of methods and measures included in comparison

On each of the artificial sequence sets, we applied our method (including scanning for TFBSs, clustering of sequences according to GC content, up to the actual prediction of regions of local enrichment), as well as a number of other methods and measures of motif enrichment.

The following is a brief description of each method and measure we included in the comparison, in addition to our own proposed method. Unless mentioned specifically, default parameters were used.

1. Method proposed by Berendzen and colleagues [11]. This is an approach for the prediction of regulatory motifs with a “positional disequilibrium”. Briefly, for each position in a set of aligned promoters, the number of TFBSs overlapping that position is converted to a Z score, reflecting the preference for a regulatory motif for that position. Positions with Z score ≥ 4 are regarded as significant.
2. Method proposed by Vardhanabhuti et al. [12]. Positional specificity is evaluated using a Z score based on a binomial distribution, with a window size of 20 bps. Parameters for the binomial distribution are based on predicted sites of permuted PWMs of the PWM of interest, thus taking into account to some extent the influence of GC content of PWMs. Originally, the authors defined every motif that has a Z-max ≥ 5 as “position-specific”. Here we slightly modified this definition in order to make direct comparison with our method possible, and considered each window with Z-score ≥ 5 as a region of local enrichment.
3. A-GLAM is a method for predicting *cis-*regulatory elements making use of their positional preferences [13, 14]. Input sequences are aligned (without gaps) by their TSSs, after which k-mers are evaluated for their consistent positioning with respect to the TSSs. The genomic set of promoter sequences was used as background model.
4. FREE is a method which uses regression analysis and a likelihood test for predicting motifs with position-specific over-representation [15]. The distribution of motif instances in a set of promoters is modeled using a so-called ‘background function’ and a ‘signal function’. A likelihood test is used to determine whether the instances of the motif can be better explained by the combining the background function (no positional bias) with the signal function (representing positional bias). The method was run using 5-mers, and a smoothing window of size 20 bps was used (option –w), as recommended. K-mers with low numbers of occurrences were excluded, but thresholds were set lower than default (-x 0.01 and –X 1).
5. TFM-Explorer circumvents problems associated with standard scanning window approaches using a heuristic algorithm that takes into account observed and expected frequencies of TFBSs, using a Poisson distribution for evaluating the significance of observed hits in candidate windows [16]. A p value cutoff of 1e-6 was used.
6. Method proposed by Casimiro and colleagues [17]. This method aims at finding positional preferences of motifs, not necessarily local enrichment. They found that their proposed “bootstrap Chi-Square test” was the most powerful test for smaller sample sites, so we employed this method in our comparison. Briefly, the observed and expected number of TFBSs in bins is compared, and a bootstrap method is used to assign a p value to the observed difference.

We also included a number of measures for global enrichment (as opposed to local enrichment) of regulatory motifs. In all cases, these were applied to the TFBSs predicted in the region -500 to +500, covering the region in which the vast majority of artificial TFBSs were inserted.

1. Over-Representation Index (ORI) analysis: a measure for global over-representation (see main manuscript). We evaluated the performance of this measure using two p value thresholds (0.01 and 0.001).
2. Binomial distribution-based enrichment: This measure for global enrichment is adopted from the method used in RSAT [18]. P values are converted to a *sig* value, with higher *sig* values representing higher enrichment. We evaluated the performance of this measure using two *sig* value thresholds (the default value 0, and 2).
3. Hypergeometric distribution as used in AlignACE [19]. The number of sequences containing at least 1 predicted TFBS in the set of sequences of interest is compared to the genomic set of promoters using a hypergeometric distribution. We evaluated the performance of this measure using two p value thresholds (0.01 and 1e-4).
4. One-tailed Fisher exact test as used in oPOSSUM [20]: this measure too compares the number of sequences in the set of interest containing at least 1 predicted TFBSs with the genomic set of sequences, and is similar to the hypergeometric probability-based method described above. We evaluated the performance of this measure using two p value thresholds (0.01 and 1e-4).
5. Information content-based measure, as used in FIRE [21]. The information content of the presence/absence of predicted TFBSs in the set of interest and the remained of the genome-wide set of sequences is calculated, and subjected to randomization tests. The observed information content is compared to 10,000 randomly selected sets of sequences. Cases where the observed information content is higher than that of all 10,000 random sets are considered as significantly over-represented.

### Measures of performance

Performances of all methods and measures were evaluated on the level of recall, precision, and F-measure, based on the numbers of true positives (TP), false negatives (FN), and false positives (FP).

Recall ( TP / (TP + FN) ) was defined as the fraction of artificially inserted regions of enrichment that could be retrieved. For methods predicting regions of preferential positioning of TFBSs or local enrichment of TFBSs, a true positive prediction was defined as an inserted region of local enrichment (center ± 2 SD) overlapping with one or more predicted regions. For methods for the prediction of global enrichment of TFBSs, any prediction of enrichment of one of the five PWMs used for generating artificial TFBSs was regarded as a true positive. False negative predictions were defined as artificially inserted regions of enrichment that could not be retrieved.

For the real data, recall was defined as the fraction of gene set – TF associations (derived from ChIP-seq data; see section “Construction of datasets based on real experimental data” above) that could be predicted correctly. Since specific positional preferences of the 25 TFs are unknown, any predicted region of local enrichment for the TF in question in the region -500 to +500 was considered as a correct prediction.

Precision ( TP / (TP + FP) ) was defined as the fraction of predicted enrichments corresponding to inserted regions of enrichment. For methods predicting regions of preferential positioning of TFBSs or local enrichment of TFBSs, any predicted region for one of the five inserted motifs overlapping with the region defined by the center of the normal distribution used for inserting sites ± 2 SD was considered as a true positive prediction. Any other predicted region was considered a false positive prediction. For methods for prediction of global enrichment of TFBSs, any prediction of enrichment of one of the five PWMs used for generating artificial TFBSs was regarded as a true positive. Any other predicted enrichment was considered a false positive prediction.

For the real data, precision was defined as the fraction of predicted enrichments that corresponded with the experimentally derived gene set – TF associations (see section “Construction of datasets based on real experimental data” above). Here too, since specific positional preferences of the 25 TFs are unknown, any predicted region of local enrichment for the TF in question in the region -500 to +500 was considered as a correct prediction. The lack of comprehensive ChIP-seq data makes it difficult to define false positive predictions. We therefore made the simplifying assumption that any predicted region of local enrichment for TFs for which no ChIP-seq data was available was a false positive. Although this is likely to result in some correct predictions being labeled as false positives, we believe it is a reasonable approximation.

F-measure, a measure for accuracy combining precision and recall, was defined as follows: F-measure = 2 x (precision x recall) / (precision + recall).

## Enrichment of weak binding sites

Weak TFBSs were defined based on the notion that PWM scores are correlated with the binding energy between TFs and DNA motifs [22, 23]: In addition to the standard PWM score threshold defined in section “Prediction of transcription factor binding sites”, a second, lower, score threshold was set in a way that results in about 1 hit per 1000 bps in the genomic set of promoter sequences. We defined weak TFBSs as sites with a score between these 2 thresholds. Using this second set of threshold scores, sites for all PWMs were predicted in the genomic set of sequences. At positions where 2 or more overlapping sites for the same PWM were found, sites with lower PWM scores were removed. This also implies that all weak TFBSs that overlap with “standard” TFBSs (e.g. sites passing the original, higher PWM score threshold) are removed. Weak sites were not used for the prediction of locally enriched regions, but were only used for the evaluation of weak TFBS over-representation.

After the prediction of locally enriched regions, for each region of local enrichment, we counted the number of weak TFBSs for the corresponding PWM within the region in all input promoter sequences. Sites overlapping stronger sites were not used. The significance of this count was evaluated using a random sampling approach. We randomly selected 10,000 sets of N sequences (See section “Random sampling approach”), and counted the number of weak sites in the region. The ratio of randomly selected sets with a count equal to or higher than the observed count in the input set of promoter sequences was used as an indicator of the significance of enrichment.

## Evolutionary conservation of predicted binding sites within locally enriched regions

Evolutionary conservation of TFBSs within locally enriched regions was evaluated using PhastCons scores as available on the UCSC Genome Browser [24]. These scores reflect the evolutionary conservation at a base-by-base level, based on multiple sequence alignments with 45 and 29 vertebrate genomes for the human and mouse genome, respectively. For each locally enriched region that contained at least 10 TFBSs, the average PhastCons score of all bases corresponding to the TFBSs in the set of input promoter sequences located within the enriched region was calculated. Since bases located proximal to TSSs tend to be more conserved than bases located at distal positions, we compared the observed PhastCons scores with those of an equal number of bases located within predicted TFBSs at equal distances to randomly selected TSSs (see “Random sampling approach”). Z scores for observed PhastCons scores were calculated based on the average and standard deviation of the PhastCons scores obtained from 100 randomly selected sets.

The significance of the number of locally enriched regions with Z scores ≥ 1 (74 out of 154 regions for the human datasets; 92 out of 205 regions for the mouse datasets) was evaluated based on a binomial distribution, with the number of trials being the total number of regions, and the probability of success being 0.159 (the probability of a Z score ≥ 1 given a normal distribution). Random samplings confirmed that this expected probability of success is accurate; 100 sets of Z scores of bases in randomly selected TFBSs followed resulted in roughly 15.9% of the sets having Z scores ≥ 1 (on average 25.0 out of 154 sets for human (16.3%); 33.2 out of 205 for mouse (16.2%) ) .

# Supplementary figures and tables

**Supplementary Figure S1: Workflow of our approach for the detection of locally over-represented TFBSs.** The analysis starts with a set of promoter regions associated with a set of genes that show a similar spatio-temporal expression profile, and the predicted binding sites for a given TF within these promoter sequences. Based on the TFBSs, the *S_local_* profiles are calculated using 5 different window function widths. In the next step the significance of the observed *S_local_* profile is estimated using a random sampling approach based on predicted TFBSs in the genome-wide set of promoter sequences, giving us a *P_dep_* and *P_ind_* value for each position in the promoters. Finally, any regions passing the p-value criteria are reduced to a non-redundant set of regions with significant local enrichment.

**Supplementary Figure S2: Illustration of the effect of the normalization factor *Z_s_*** **on the local enrichment scores.** (A) A toy example of 5 promoter sequences with predicted binding sites for a transcription factor indicated by blue boxes. Sequence 5 contain a distal cluster of 4 predicted sites, possibly caused by low complexity repeat sequences. (B) Contributions *p_s_* of each sequence to the *S_local_*, and at the bottom the *S_local_* calculated with and without the normalization factor *Z_s_*. Note that the scale of the Y axis is different for sequence 5. When the normalization factor *Z_s_* is not used, the distal cluster of sites results in a strong peak in *S_local_*, even though only 1 of 5 sequences contains predicted sites in this region. When using the normalization factor, this peak disappears and only the proximal peak remains. This peak is supported by predicted sites in each of the 5 sequences and thus more likely to be biologically relevant.

**Supplementary Figure S3: GC content profiles of clusters obtained by k-means clustering of the genome-wide set of human promoter sequences, with *k* values 1 to 6.** For each k-means clustering the average GC content of the promoter sequences in each cluster is shown in bins of 100 bps in the region -1kb to +1kb. Graphs A to F correspond to the cases with *k* values from 1 to 6, respectively. The graph for *k* = 1 shows the average GC content of the genome-wide set of promoter sequences.

**Supplementary Figure S4: Workflow of the standard TFBS over-representation analysis used in this study.** The general flow of this approach is similar to the one for the detection of locally enriched TFBSs. The main difference is that instead of the *S_local_* profiles, a single parameter for over-representation is calculated; the *ORI* value. The significance of this ORI value is evaluated using a random sampling approach, giving us a *P_ORI_* value. Motifs having a *P_ORI_* value below the p-value threshold are considered to be “globally” over-represented.

**Supplementary Figure S5: General trends of significantly locally enriched regions detected in the GNF GeneAtlas mouse gene sets.** These figures are the counterparts of Fig. 3 B and C in the main manuscript, for mouse gene sets. (A) Mouse enriched regions sorted by Z score of PhastCons scores of the TFBSs predicted within each region. (B) Mouse enriched regions sorted by p-value of enrichment of weak TFBSs within each region.

**Supplementary Table S1: Annotation of sets of co-regulated genes of the mouse GNF GeneAtlas data.** For each set of co-regulated genes the tissues in which the highest expression was observed is listed, along with the number of promoter sequences it contains, and the *k** value (see Methods).
**Note:** in cases where multiple clusters of genes are associated with similar tissues or cell types, expression levels of the genes in these clusters are different in other tissues or cell types.

| **Cluster index** | **Main tissues or cell types associated with this cluster** | **Number of sequences** | ***k**** |
| --- | --- | --- | --- |
| 1 | Embryo | 178 | 2 |
| 2 | B220 B cell, CD4+ T cell, CD8+ T cell, thymus | 277 | 2 |
| 3 | thyroid, Salivary gland | 47 | 2 |
| 4 | skeletal muscle, heart, kidney, snout epidermis, adrenal gland | 210 | 3 |
| 5 | preoptic, hypothalamus, substantia nigra, amygdala, olfactory bulb | 385 | 3 |
| 6 | thymus, ovary, B220 B cell, embryo | 292 | 2 |
| 7 | adipose tissue, liver, kidney, adrenal gland, brownfat | 296 | 2 |
| 8 | Placenta | 93 | 2 |
| 9 | tongue epidermis, snout epidermis, digits | 122 | 2 |
| 10 | Testis | 190 | 2 |
| 11 | dorsal root ganglion, trigeminal | 71 | 2 |
| 12 | CD4+ T cell, CD8+ T cell, B220 B cell | 221 | 2 |
| 13 | dorsal root ganglion, dubstantia nigra, preoptic, hypothalamus, trigeminal | 255 | 3 |
| 14 | oocyte, fertilized egg | 282 | 2 |
| 15 | kidney, liver | 158 | 2 |
| 16 | liver | 150 | 2 |
| 17 | amygdala, frontal cortex, cortex, olfactory bulb, hippocampus | 378 | 4 |
| 18 | lung | 96 | 2 |
| 19 | small intestine, large intestine | 159 | 2 |
| 20 | umbilical cord | 396 | 4 |
| 21 | retina | 85 | 2 |
| 22 | testis | 520 | 2 |
| 23 | oocyte, fertilized egg | 134 | 2 |
| 24 | oocyte, fertilized egg | 313 | 2 |
| 25 | oocyte, fertilized egg | 483 | 2 |
| 26 | snout epidermis, digits | 225 | 2 |
| 27 | bone, bone marrow | 101 | 2 |
| 28 | B220 B cell, thymus, CD4+ T cell, trachea, CD8+ T cell | 264 | 2 |
| 29 | B220 B cell, lymph node | 79 | 2 |
| 30 | adrenal gland, adipose tissue, ovary, dorsal root ganglion, snout epidermis | 228 | 3 |
| 31 | prostate | 169 | 2 |
| 32 | cerebellum, preoptic, hypothalamus, substantia nigra, spinal cord lower | 188 | 2 |
| 33 | blastocysts | 178 | 2 |
| 34 | oocyte, fertilized egg | 158 | 2 |
| 35 | testis | 188 | 4 |
| 36 | pancreas, spleen | 52 | 3 |
| 37 | oocyte, fertilized egg, B220 B cell, thymus, CD4+ T cell | 158 | 2 |
| 38 | B220 B cell, CD4+ T cell, thymus, CD8+ T cell, lymph node | 97 | 2 |
| 39 | skeletal muscle, heart | 112 | 2 |
| 40 | mammary gland (lact), adrenal gland, ovary | 143 | 2 |
| 41 | thymus | 61 | 2 |
| 42 | skeletal muscle | 101 | 2 |
| 43 | stomach | 61 | 2 |
| 44 | medial olfactory epithelium, vomeral nasal organ | 89 | 2 |

**Supplementary Table S2: Annotation of sets of co-regulated genes of the human GNF GeneAtlas data.** For each set of co-regulated genes the tissues in which the highest expression was observed is listed, along with the number of promoter sequences it contains, and the *k** value (see Methods).
**Note:** See note of Supplementary Table S1.

| **Cluster index** | **Main tissues or cell types associated with this cluster** | **Number of sequences** | ***k**** |
| --- | --- | --- | --- |
| 1 | brain amygdala, Whole brain, Prefrontal cortex, cerebellum, occipital lobe | 407 | 3 |
| 2 | 721 B-lymphoblasts, BM CD105+ endothelial cells | 205 | 6 |
| 3 | BM CD33+ myeloid cells, PB CD14+ monocytes, whole blood | 407 | 2 |
| 4 | thyroid, heart, skeletal muscle psoas, tongue, fetal thyroid | 273 | 2 |
| 5 | PB CD8+ T cells, PB CD56+ NK cells, PB CD4+ T cells, PB CD19+ B cells, PB BDCA-4 dentritic cells | 574 | 2 |
| 6 | PB CD8+ T cells, PB CD4+ T cells, PB CD56+ NK cells | 202 | 2 |
| 7 | liver, fetalliver, kidney | 280 | 2 |
| 8 | lung, uterus, prostate, trachea, human cultured adipocyte | 370 | 4 |
| 9 | smooth muscle, cardiacmyocytes | 188 | 3 |
| 10 | superior cervical ganglion, skeletal muscle psoas, appendix | 661 | 2 |
| 11 | 721 B-lymphoblasts, BM CD34+ cells, whole brain, PB BDCA-4 dentritic cells, PB CD56+ NK cells | 561 | 3 |
| 12 | 721 B-lymphoblasts, BM CD34+ cells, BM CD105+ endothelial cells, PB BDCA-4 dentritic cells, PB CD56+ NK cells | 793 | 5 |
| 13 | PB CD19+ B cells, Lymphoma Burkitts, BM CD34+ cells, PB CD4+ T cells, PB CD8+ T cells | 140 | 2 |
| 14 | BM CD34+ cells, 721 B-lymphoblasts, PB CD56+ NK cells, PB BDCA-4 dentritic cells, PB CD19+ B cells | 726 | 6 |
| 15 | PB CD56+ NK cells, PB CD8+ T cells, PB BDCA-4 dentritic cells, 721 B-lymphoblasts, BM CD34+ cells | 476 | 2 |
| 16 | smooth muscle, placenta, lung, PB CD14+ monocytes, BM CD33+ myeloid cells | 452 | 2 |
| 17 | human bronchial epithelial cells, colorectal adenocarcinoma | 166 | 4 |
| 18 | testis | 157 | 2 |
| 19 | BM CD71+ early erythroid cells | 391 | 4 |
| 20 | prefrontal cortex, brain amygdala, hypothalamus, whole brain, occipital lobe | 479 | 2 |
| 21 | 721 B-lymphoblasts | 227 | 2 |
| 22 | PB BDCA-4 dentritic cells, PB CD19+ B cells | 357 | 2 |
| 23 | brain amygdala, whole brain, prefrontal cortex, occipital lobe, hypothalamus | 691 | 3 |
| 24 | placenta | 128 | 4 |
| 25 | tongue, adrenal gland, adrenal cortex, skin, superior cervical ganglion | 262 | 2 |
| 26 | BM CD71+ early erythroid cells, PB CD14+ monocytes, colorectal adenocarcinoma, PB BDCA-4 dentritic cells, BM CD105+ endothelial cells | 88 | 4 |
| 27 | globus pallidus, subthalamic nucleus, brain thalamus, ciliary ganglion, atrioventricular node | 122 | 4 |
| 28 | superior cervical ganglion, appendix, atrioventricular node, drg, ciliary ganglion | 116 | 4 |
| 29 | pancreas, Islet cell | 50 | 2 |
| 30 | testis | 403 | 2 |
| 31 | skin, atrioventricular node, trigeminal ganglion, PB CD8+ T cells, ciliary ganglion | 32 | 2 |
| 33 | brain caudate nucleus, cardiac myocytes, globus pallidus, subthalamic nucleus, pons | 31 | 4 |

**Note:** The cluster with cluster index 32 is not included in the table. It was not used for analysis because it contained only 2 sequences.

**Supplementary Table S3: Annotation of sets of co-regulated genes of the Amit *et al*. mouse DC data.** For each set of co-regulated genes a brief description of their expression profile is given, along with the number of promoter sequences it contains, and the *k** value (see Methods).

| **Cluster index** | **Description of expression profile** | **Number of sequences** | ***k**** |
| --- | --- | --- | --- |
| 1 | transient upregulation, weak in Pic | 128 | 3 |
| 2 | transient downregulation, strong in LPS | 189 | 3 |
| 3 | transient downregulation, weak in Pic | 126 | 3 |
| 4 | late upregulation, not in Pic | 52 | 2 |
| 5 | transient downregulation, strong in LPS | 72 | 2 |
| 6 | downregulation | 103 | 2 |
| 7 | downregulation, not in Pam | 87 | 2 |
| 8 | downregulation, not in Pic, Controls | 62 | 2 |
| 9 | Peak at 60, 120 min, not in Pic | 83 | 3 |
| 10 | strong downregulation at 30 min | 84 | 2 |
| 11 | late downregulation | 72 | 2 |
| 12 | transient upregulation, not in Pam, not transient in Pic | 153 | 2 |
| 13 | upregulated in Pic, weak in LPS | 67 | 2 |
| 14 | upregulated, not in Pic | 93 | 3 |
| 15 | transient upregulation, strong in LPS | 89 | 3 |
| 16 | continuous upregulation, not in Pic | 78 | 2 |
| 17 | upregulated, from about 240 min | 45 | 3 |
| 18 | transient upregulation, weak in CpG, Grd, not in Pam | 92 | 2 |

**Supplementary Table S4: Annotation of 6 sets of co-regulated, inducible genes of the Garber *et al.* data, used for the comparison of the performance.** For each set of co-regulated genes a brief description of their expression profile is given, along with the number of promoter sequences it contains, and the *k** value (see Methods).

| **Cluster index** | **Description of expression profile** | **Number of sequences** | ***k**** |
| --- | --- | --- | --- |
| 1 | late induction (4h) | 321 | 2 |
| 2 | induction at 2h after stimulation | 313 | 4 |
| 3 | early induction (1h) | 177 | 3 |
| 4 | transient early induction (1h) | 129 | 3 |
| 5 | transient early induction (2h) | 136 | 4 |
| 6 | strong, late induction (4h) | 207 | 2 |

**Supplementary Table S5: Full overview of results for the mouse GNF GeneAtlas gene sets.** (see Excel file) The table gives for each motif and each cluster of promoters the regions of local enrichment (if any, at the top) and the *P_ORI_* values (at the bottom). These are the raw results of our analysis and are given mainly for completeness.

**Supplementary Table S6: Full overview of results for the human GNF GeneAtlas gene sets.** (see Excel file) The table gives for each motif and each cluster of promoters the regions of local enrichment (if any, at the top) and the *P_ORI_* values (at the bottom). These are the raw results of our analysis and are given mainly for completeness.

**Supplementary Table S7: A selection of predicted regions of local enrichment that could also be found using standard TFBS over-representation analysis.** Regulatory motifs are shown for which regions of local enrichment were detected in mouse and human tissues and cell types of the GNF GeneAtlas dataset. The species, cluster index, tissues and cell types, TF or motif name, the start and stop position of the region, the $h/2$ used, the ORI p-value, and references supporting the role of the regulatory motif in the tissue in question are shown.

| **Species** | **Cluster index** | **Main tissues or cell types associated with this cluster** | **Motif** | **Region of enrichment: x_1_ to x_2_ (**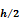**)** | ***P_ORI_*** | **References** |
| --- | --- | --- | --- | --- | --- | --- |
| mouse | 2 | B cells, T cells | ETS family of TFs, and SH2D1A | -106 to 17 (50) | **0.0000** | [25] |
| mouse | 2 | B cells, T cells | RBPJ | -139 to -70 (100) | **0.0057** | [26, 27] |
| mouse | 10 | testis | RFX factors | -128 to 65 (50) | **0.0000** | [28, 29] |
| mouse | 10 | testis | MYB family TFs | -214 to 113 (200) | **0.0007** | [30, 31] |
| mouse | 10 | testis | NRF-1 | -143 to 38 (200) | **0.0012** | [32] |
| mouse | 12 | T cells, B cells | NF-kappaB | -309 to -100 (200) | **0.0000** | [33] |
| mouse | 12 | T cells, B cells | ETS domain TFs, including SPI1 or PU.1 | -232 to 47 (100) | **0.0000** | [34, 35] |
| mouse | 12 | T cells, B cells | RUNX TFs | -142 to -23 (50) | **0.0002** | [36, 37] |
| mouse | 12 | T cells, B cells | MZF1 | -155 to 119 (200) | **0.0011** | [38, 39] |
| mouse | 12 | T cells, B cells | IRF FAMILY | -119 to 6 (50) | **0.0047** | [40] |
| mouse | 12 | T cells, B cells | ETS family of TFs | -133 to -98 (100) | **0.0073** | [25] |
| mouse | 15 | kidney, liver | HNF1 | -125 to -24 (20) | **0.0000** | [41, 42] |
| mouse | 15 | kidney, liver | Nuclear receptors, HNF4 | -401 to -66 (200) | **0.0000** | [43, 44] |
| mouse | 15 | kidney, liver | HNF4 | -105 to -41 (20) | **0.0001** | [45, 46] |
| mouse | 15 | kidney, liver | Ikaros | -511 to -250 (200) | **0.0001** | [47] |
| mouse | 15 | kidney, liver | HNF4A | -122 to -24 (50) | **0.0003** | [45, 46] |
| mouse | 16 | liver | HNF1 | -92 to -27 (20) | **0.0000** | [41, 42] |
| mouse | 16 | liver | Lmo2 complex, GATA1, 3 and 6 | -93 to -30 (100) | **0.0002** | [48, 49] |
| mouse | 16 | liver | HNF4 | -94 to -28 (50) | **0.0003** | [45, 46] |
| mouse | 16 | liver | HNF4A | -177 to -15 (100) | **0.0015** | [45, 46] |
| mouse | 19 | small intestine, large intestine | Nuclear receptors, HNF4 | -112 to -68 (50) | **0.0023** | [50] |
| mouse | 19 | small intestine, large intestine | HNF4 | -120 to 93 (200) | **0.0026** | [51-53] |
| mouse | 19 | small intestine, large intestine | HNF4A | -215 to -17 (100) | **0.0037** | [51-53] |
| mouse | 22 | testis | CREB-binding TFs, including ATF family | -93 to -16 (20) | **0.0000** | [54, 55] |
| mouse | 22 | testis | RFX factors | -238 to 86 (100) | **0.0000** | [28, 29] |
| mouse | 22 | testis | RFX1 | -156 to 36 (100) | **0.0002** | [28, 29] |
| mouse | 22 | testis | RFX TFS | -153 to 47 (200) | **0.0005** | [28, 29] |
| mouse | 22 | testis | MYB family TFs | -167 to 147 (100) | **0.0006** | [30, 31] |
| mouse | 42 | skeletal muscle | EGR factors | -250 to -29 (200) | **0.0006** | [56-59] |
| human | 7 | liver, fetal liver, liver | HNF1 | -175 to -1 (50) | **0.0000** | [41, 42] |
| human | 7 | liver, fetal liver, liver | HNF4 | -378 to 150 (200) | **0.0000** | [45, 46] |
| human | 7 | liver, fetal liver, liver | Nuclear receptors, HNF4 | -115 to -25 (50) | **0.0000** | [43, 44] |
| human | 7 | liver, fetal liver, liver | HNF4A | -204 to -10 (100) | **0.0000** | [45, 46] |
| human | 11 | 721 B-lymphoblasts, BM CD34+ cells | ETS family of TFs | -174 to 42 (100) | **0.0000** | [25] |
| human | 11 | 721 B-lymphoblasts, BM CD34+ cells | ZFX | 121 to 279 (100) | **0.0004** | [60] |
| human | 11 | 721 B-lymphoblasts, BM CD34+ cells | MTF1 | -231 to -90 (200) | **0.0079** | [61] |
| human | 12 | 721 B-lymphoblasts, BM CD34+ cells | ETS family of TFs | -151 to 37 (100) | **0.0000** | [25] |
| human | 12 | 721 B-lymphoblasts, BM CD34+ cells | MYB family TFs | -162 to -32 (100) | **0.0002** | [62] |
| human | 12 | 721 B-lymphoblasts, BM CD34+ cells | STAT family | -187 to -163 (200) | **0.0005** | [63] |
| human | 12 | 721 B-lymphoblasts, BM CD34+ cells | Whn (= FOXN1, nude) | -129 to 19 (100) | **0.0006** | [64, 65] |
| human | 12 | 721 B-lymphoblasts, BM CD34+ cells | YY1 | -80 to 111 (100) | **0.0059** | [66, 67] |
| human | 13 | B cells, Burkitt's lymphoma | ETS family of TFs | -148 to 23 (100) | **0.0000** | [25] |
| human | 13 | B cells, Burkitt's lymphoma | MYB family TFs | -834 to -765 (50) | **0.0002** | [62] |
| human | 14 | BM CD34+ cells, 721 B-lymphoblasts | ETS family of TFs | -81 to 10 (50) | **0.0000** | [25] |
| human | 14 | BM CD34+ cells, 721 B-lymphoblasts | CREB-binding TFs, including ATF family | -84 to -78 (50) | **0.0017** | [68-71] |
| human | 15 | NK cells, T cells | ETS family of TFs | -97 to 34 (50) | **0.0000** | [25] |
| human | 15 | NK cells, T cells | YY1 | -82 to 112 (100) | **0.0046** | [67] |
| human | 18 | testis | NRF-1 | -164 to -42 (100) | **0.0029** | [32] |
| human | 18 | testis | CREB-binding TFs | -255 to -110 (200) | **0.0050** | [54, 55] |
| human | 30 | testis | CREB-binding TFs, including ATF family | -113 to -9 (50) | **0.0000** | [54, 55] |
| human | 30 | testis | MYB family TFs | -210 to 117 (100) | **0.0000** | [30, 31] |
| human | 30 | testis | RFX1 | -34 to 49 (100) | **0.0048** | [28, 29] |

**Supplementary Table S8: A selection of predicted regions of local enrichment in the mouse GNF GeneAtlas datasets that could not be found using standard TFBS over-representation analysis.** For a number of mouse tissues and cell types included in the GNF GeneAtlas dataset, the tables shows regulatory motifs for which a region of local enrichment of sites was detected, with the tissue in which it was found, the start and stop position of the region, the
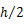
 used, the *ORI* p-value (as measure of over-representation in the region -1 kb to -1), and references supporting the importance of the regulatory motif in the tissue in question. Only regions of enrichment located inside the region -500 to +500 around the TSS are shown. Table 1 in the main paper shows a subset of these results.

| **Cluster index** | **Main tissues or cell types associated with this cluster** | **Motif** | **Region of enrichment:  x_1_ to x_2_ (**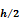**)** | ***P_ORI_*** | **References** |
| --- | --- | --- | --- | --- | --- |
| 2 | B cells, T cells | HIF1 | 55 to 200 (200) | 0.1093 | [72] |
| 2 | B cells, T cells | ETS domain TFs, including SPI1 or PU.1 | -10 to 0 (10) | 0.1529 | [35, 73] |
| 10 | testis | RFX1 | -91 to 129 (200) | 0.0116 | [28, 29] |
| 10 | testis | CREB-binding TFs, including ATF family | -148 to 31 (100) | 0.0350 | [54, 55] |
| 10 | testis | RFX TFs | -61 to 35 (50) | 0.2589 | [28, 29] |
| 12 | T cells, B cells | GC-box, Zinc finger TFs (Sp TFs, KLF TFs) | -193 to -82 (100) | 0.0295 | [74, 75] |
| 16 | liver | Cux1 (CR3+HD) | -103 to -90 (10) | 0.0374 | [76] |
| 19 | small intestine, large intestine | HNF1 | -93 to -37 (20) | 0.0115 | [77] |
| 19 | small intestine, large intestine | RXR, RAR, and VDR | -52 to -43 (10) | 0.0654 | [78, 79] |
| 22 | testis | MYB family TFs | -72 to 95 (100) | 0.0245 | [30, 31] |
| 22 | testis | heat shock factors | -58 to 217 (200) | 0.0317 | [80] |
| 22 | testis | PPARA, RXRA, VDR, AIRE | -44 to -18 (20) | 0.1159 | [81, 82] |
| 42 | skeletal muscle | THR alpha and beta | -30 to -15 (50) | 0.0248 | [83-86] |

**Supplementary Table S9: A selection of predicted regions of local enrichment in the human GNF GeneAtlas datasets that could not be found using standard TFBS over-representation analysis.** For a number of human tissues and cell types included in the GNF GeneAtlas dataset, the tables shows regulatory motifs for which a region of local enrichment of TFBSs was detected, with the tissue in which it was found, the start and stop position of the region, the
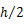
 used, the *ORI* p-value (as measure of over-representation in the region -1 kb to -1), and references supporting the importance of the regulatory motif in the tissue in question. Only regions of enrichment located inside the region -500 to +500 around the TSS are shown.

| **Cluster index** | **Main tissues or cell types associated with this cluster** | **Motif** | **Region of enrichment: x_1_ to x_2_ (**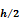**)** | ***P_ORI_*** | **References** |
| --- | --- | --- | --- | --- | --- |
| 7 | liver, fetal liver, liver | Sp1 | -105 to -84 (50) | 0.0111 | [87, 88] |
| 7 | liver, fetal liver, liver | ZIC and GLI zinc finger TFs | -192 to -31 (200) | 0.0175 | [89] |
| 7 | liver, fetal liver, liver | AR | -200 to -2 (200) | 0.0292 | [90] |
| 11 | 721 B-lymphoblasts, BM CD34+ cells | HIF1 | -41 to 194 (200) | 0.0229 | [72] |
| 11 | 721 B-lymphoblasts, BM CD34+ cells | YY1 | -81 to 123 (100) | 0.0319 | [66, 67] |
| 12 | 721 B-lymphoblasts, BM CD34+ cells | Nkx2-3, Nkx2-9, Nkx3-1 | 926 to 1000 (200) | 0.6105 | [91, 92] |
| 13 | B cells, Burkitt's lymphoma | Klf4 | -8 to 6 (10) | 0.0462 | [93-95] |
| 13 | B cells, Burkitt's lymphoma | ZEB1 | -40 to -30 (20) | 0.0573 | [96, 97] |
| 13 | B cells, Burkitt's lymphoma | ETS domain TFs, including SPI1 or PU.1 | -66 to 32 (50) | 0.0577 | [35, 73] |
| 13 | B cells, Burkitt's lymphoma | YY1 | -63 to 174 (100) | 0.4782 | [66, 67, 98] |
| 13 | B cells, Burkitt's lymphoma | E box-binding TFs | 248 to 578 (200) | 0.9940 | [99, 100] |
| 14 | BM CD34+ cells, 721 B-lymphoblasts | ETS domain TFs, including SPI1 or PU.1 | -81 to -32 (50) | 0.0651 | [35, 73] |
| 14 | BM CD34+ cells, 721 B-lymphoblasts | YY1 | -36 to 52 (100) | 0.4645 | [66, 67] |
| 15 | NK cells, T cells | Whn | -125 to 23 (100) | 0.0375 | [101, 102] |
| 18 | Testis | MYB family TFs | -19 to 3 (20) | 0.1184 | [30, 31] |
| 18 | Testis | BLIMP1 (PRDM1) | 228 to 255 (50) | 0.5756 | [103, 104] |
| 30 | Testis | RFX factors | -140 to 87 (100) | 0.0135 | [28, 29] |
| 30 | Testis | AHR, ARNT | -158 to -158 (200) | 0.0546 | [105] |

**Supplementary Table S10: Examples of TFBSs for which regions of local enrichment were detected in different sets of sequences.** We list a number of TFs whose binding motifs showed significant local enrichment in multiple sets of promoter sequences. The species, and the tissues and cell lines associated with the promoters in which the motif was found are listed, as well as the regions of local enrichment. Only regions of enrichment located inside the region -500 to +500 around the TSS are shown.

| **TF or regulatory motif** | **Species** | **Cluster index** | **Main tissues or cell types associated with this cluster** | **Region of enrichment: x_1_ to x_2_ (**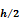**)** |
| --- | --- | --- | --- | --- |
| ETS family of TFs | human | 5 | T cells, NK cells | -141 to 51 (100) |
|  |  | 11 | 721 B-lymphoblasts, BM CD34+ cells | -174 to 42 (100) |
|  |  | 12 | 721 B-lymphoblasts, BM CD34+ cells | -151 to 37 (100) |
|  |  | 13 | B cells, Burkitt's lymphoma | -148 to 23 (100) |
|  |  | 14 | BM CD34+ cells, 721 B-lymphoblasts | -81 to 10 (50) |
|  |  | 15 | NK cells, T cells | -97 to 34 (50) |
|  | mouse | 2 | B cells, T cells | -106 to 17 (50) |
|  |  | 4 | Skeletal muscle, heart | -161 to 26 (100) |
|  |  | 6 | thymus, ovary | -137 to 18 (100) |
|  |  | 10 | testis | -133 to -58 (100) |
|  |  | 12 | T cells, B cells | -133 to -98 (100) |
|  |  | 25 | oocyte, fertilized egg | -139 to 42 (100) |
|  |  | 34 | oocyte, fertilized egg | -240 to 34 (200) |
|  |  | 35 | testis | -178 to 71 (200) |
|  |  | 37 | oocyte, fertilized egg | -77 to -11 (50) |
| YY1 | human | 11 | 721 B-lymphoblasts, BM CD34+ cells | -81 to 123 (100) |
|  |  | 12 | 721 B-lymphoblasts, BM CD34+ cells | -80 to 111 (100) |
|  |  | 13 | B cells, Burkitt's lymphoma | -63 to 174 (100) |
|  |  | 14 | BM CD34+ cells, 721 B-lymphoblasts | -36 to 52 (100) |
|  |  | 15 | NK cells, T cells | -82 to 112 (100) |
|  |  | 20 | prefrontal cortex, brain amygdala | -46 to 217 (200) |
|  | mouse | 2 | B cells, T cells | -63 to 153 (100) |
|  |  | 4 | Skeletal muscle, heart | -40 to 85 (50) |
|  |  | 6 | thymus, ovary | -61 to 122 (100) |
| NRF-1 | human | 2 | 721 B-lymphoblasts, BM CD105+ endothelial cells | -123 to -88 (100) |
|  |  | 11 | 721 B-lymphoblasts, BM CD34+ cells | -127 to -3 (100) |
|  |  | 12 | 721 B-lymphoblasts, BM CD34+ cells | -109 to -77 (100) |
|  |  | 18 | Testis | -164 to -42 (100) |
|  | mouse | 4 | Skeletal muscle, heart | -153 to -59 (100) |
|  |  | 6 | thymus, ovary | -125 to -38 (100) |
|  |  | 10 | Testis | -143 to 38 (200) |
|  |  | 25 | oocyte, fertilized egg | -104 to -20 (50) |
|  |  | 37 | oocyte, fertilized egg | -81 to -65 (20) |
| HNF4A | human | 7 | liver, fetal liver, liver | -204 to -10 (100) |
|  |  | 15 | kidney, liver | -122 to -24 (50) |
|  |  | 16 | liver | -177 to -15 (100) |
|  |  | 19 | small intestine, large intestine | -215 to -17 (100) |
| Sp1 | human | 7 | liver, fetal liver, liver | -105 to -84 (50) |
|  |  | 16 | smooth muscle, placenta | -124 to -52 (50) |
|  | mouse | 7 | adipose tissue, liver | -115 to -25 (50) |
|  |  | 20 | umbilical cord | -76 to -72 (100) |
|  |  | 24 | oocyte, fertilized egg | -86 to 0 (200) |
|  |  | 28 | B cell, thymus | -146 to -37 (100) |
|  |  | 33 | blastocysts | -159 to -78 (100) |
| RFX1 | human | 30 | testis | -34 to 49 (100) |
|  | mouse | 10 | testis | -91 to 129 (200) |
|  |  | 14 | oocyte, fertilized egg | -134 to -134 (20) |
|  |  | 22 | testis | -156 to 36 (100) |
|  |  | 35 | testis | 253 to 261 (10) |
| CACCC box-binding factors | human | 8 | lung, uterus | -142 to 31 (100) |
|  |  | 10 | superior cervical ganglion, skeletal muscle psoas | -173 to -145 (100) |
|  |  | 17 | human bronchial epithelial cells, colorectal adenocarcinoma | -133 to -80 (50) |
|  | mouse | 9 | tongue epidermis, snout epidermis | -178 to -66 (50) |
|  |  | 12 | T cells, B cells | -97 to -39 (200) |
|  |  | 18 | lung | -127 to 33 (100) |
|  |  | 41 | thymus | -149 to -17 (100) |
| CREB-binding TFs, including ATF family | human | 14 | BM CD34+ cells, 721 B-lymphoblasts | -84 to -78 (50) |
|  |  | 23 | brain amygdala, whole brain | -210 to -147 (200) |
|  |  | 30 | testis | -113 to -9 (50) |
|  | mouse | 6 | thymus, ovary | -236 to -54 (200) |
|  |  | 10 | testis | -148 to 31 (100) |
|  |  | 22 | testis | -93 to -16 (20) |
| ZFX | human | 3 | BM CD33+ myeloid cells, PB CD14+ monocytes | -77 to -51 (20) |
|  |  | 11 | 721 B-lymphoblasts, BM CD34+ cells | 121 to 279 (100) |
|  |  | 12 | 721 B-lymphoblasts, BM CD34+ cells | 203 to 262 (50) |
|  | mouse | 6 | thymus, ovary | 152 to 334 (100) |
|  |  | 25 | oocyte, fertilized egg | 54 to 262 (100) |

**Supplementary Table S11: Full overview of results for the gene sets obtained from TLR-stimulated DCs.** (see Excel file) The table gives for each motif and each cluster of promoters the regions of local enrichment (if any, at the top) and the *P_ORI_* values (at the bottom). These are the raw results of our analysis and are given mainly for completeness.

# References

1. Su AI, Wiltshire T, Batalov S, Lapp H, Ching KA, Block D, Zhang J, Soden R, Hayakawa M, Kreiman G, et al: **A gene atlas of the mouse and human protein-encoding transcriptomes.** *Proc Natl Acad Sci U S A* 2004, **101:**6062-6067.

2. Amit I, Garber M, Chevrier N, Leite AP, Donner Y, Eisenhaure T, Guttman M, Grenier JK, Li WB, Zuk O, et al: **Unbiased Reconstruction of a Mammalian Transcriptional Network Mediating Pathogen Responses.** *Science* 2009, **326:**257-263.

3. Yamashita R, Wakaguri H, Sugano S, Suzuki Y, Nakai K: **DBTSS provides a tissue specific dynamic view of Transcription Start Sites.** *Nucleic Acids Res* 2010, **38:**D98-D104.

4. Carninci P, Kasukawa T, Katayama S, Gough J, Frith MC, Maeda N, Oyama R, Ravasi T, Lenhard B, Wells C, et al: **The transcriptional landscape of the mammalian genome.** *Science* 2005, **309:**1559-1563.

5. Rhead B, Karolchik D, Kuhn RM, Hinrichs AS, Zweig AS, Fujita PA, Diekhans M, Smith KE, Rosenbloom KR, Raney BJ, et al: **The UCSC Genome Browser database: update 2010.** *Nucleic Acids Research* 2010, **38:**D613-619.

6. Vandenbon A, Nakai K: **Modeling tissue-specific structural patterns in human and mouse promoters.** *Nucleic Acids Research* 2010, **38:**17-25.

7. Matys V, Kel-Margoulis OV, Fricke E, Liebich I, Land S, Barre-Dirrie A, Reuter I, Chekmenev D, Krull M, Hornischer K, et al: **TRANSFAC and its module TRANSCompel: transcriptional gene regulation in eukaryotes.** *Nucleic Acids Res* 2006, **34:**D108-110.

8. Bryne JC, Valen E, Tang MHE, Marstrand T, Winther O, da Piedade I, Krogh A, Lenhard B, Sandelin A: **JASPAR, the open access database of transcription factor-binding profiles: new content and tools in the 2008 update.** *Nucleic Acids Research* 2008, **36:**D102-D106.

9. Gupta S, Stamatoyannopoulos JA, Bailey TL, Noble WS: **Quantifying similarity between motifs.** *Genome Biol* 2007, **8:**R24.

10. Garber M, Yosef N, Goren A, Raychowdhury R, Thielke A, Guttman M, Robinson J, Minie B, Chevrier N, Itzhaki Z, et al: **A high-throughput chromatin immunoprecipitation approach reveals principles of dynamic gene regulation in mammals.** *Mol Cell* 2012, **47:**810-822.

11. Berendzen KW, Stuber K, Harter K, Wanke D: **Cis-motifs upstream of the transcription and translation initiation sites are effectively revealed by their positional disequilibrium in eukaryote genomes using frequency distribution curves.** *BMC Bioinformatics* 2006, **7:**522.

12. Vardhanabhuti S, Wang J, Hannenhalli S: **Position and distance specificity are important determinants of cis-regulatory motifs in addition to evolutionary conservation.** *Nucleic Acids Res* 2007, **35:**3203-3213.

13. Tharakaraman K, Bodenreider O, Landsman D, Spouge JL, Marino-Ramirez L: **The biological function of some human transcription factor binding motifs varies with position relative to the transcription start site.** *Nucleic Acids Research* 2008, **36:**2777-2786.

14. Kim NK, Tharakaraman K, Marino-Ramirez L, Spouge JL: **Finding sequence motifs with Bayesian models incorporating positional information: an application to transcription factor binding sites.** *BMC Bioinformatics* 2008, **9**.

15. Yokoyama KD, Ohler U, Wray GA: **Measuring spatial preferences at fine-scale resolution identifies known and novel cis-regulatory element candidates and functional motif-pair relationships.** *Nucleic Acids Research* 2009, **37:**-.

16. Defrance M, Touzet H: **Software - Predicting transcription factor binding sites using local over-representation and comparative genomics.** *BMC Bioinformatics* 2006, **7:**-.

17. Casimiro AC, Vinga S, Freitas AT, Oliveira AL: **An analysis of the positional distribution of DNA motifs in promoter regions and its biological relevance.** *BMC Bioinformatics* 2008, **9:**89.

18. van Helden J: **Regulatory Sequence Analysis Tools.** *Nucleic Acids Research* 2003, **31:**3593-3596.

19. Hughes JD, Estep PW, Tavazoie S, Church GM: **Computational identification of cis-regulatory elements associated with groups of functionally related genes in Saccharomyces cerevisiae.** *J Mol Biol* 2000, **296:**1205-1214.

20. Sui SJH, Mortimer JR, Arenillas DJ, Brumm J, Walsh CJ, Kennedy BP, Wasserman WW: **oPOSSUM: identification of over-represented transcription factor binding sites in co-expressed genes.** *Nucleic Acids Research* 2005, **33:**3154-3164.

21. Elemento O, Slonim N, Tavazoie S: **A universal framework for regulatory element discovery across all genomes and data types.** *Mol Cell* 2007, **28:**337-350.

22. Stormo GD, Zhao Y: **Determining the specificity of protein-DNA interactions.** *Nat Rev Genet* 2010, **11:**751-760.

23. Stormo GD, Fields DS: **Specificity, free energy and information content in protein-DNA interactions.** *Trends Biochem Sci* 1998, **23:**109-113.

24. Siepel A, Bejerano G, Pedersen JS, Hinrichs AS, Hou MM, Rosenbloom K, Clawson H, Spieth J, Hillier LW, Richards S, et al: **Evolutionarily conserved elements in vertebrate, insect, worm, and yeast genomes.** *Genome Research* 2005, **15:**1034-1050.

25. Macleod K, Leprince D, Stehelin D: **The Ets Gene Family.** *Trends Biochem Sci* 1992, **17:**251-256.

26. Honjo T, Han H, Tanigaki K, Yamamoto N, Kuroda K, Yoshimoto M, Nakahata T, Ikuta K: **Inducible gene knockout of transcription factor recombination signal binding protein-J reveals its essential role in T versus B lineage decision.** *Int Immunol* 2002, **14:**637-645.

27. Honjo T, Tanigaki K, Kuroda K, Han H: **Regulation of B cell development by Notch/RBP-J signaling.** *Semin Immunol* 2003, **15:**113-119.

28. Miki Y, Morotami-Yano K, Yano K, Saito H, Sun Z, Iwama A: **Human regulatory factor X 4 (RFX4) is a testis-specific dimeric DNA-binding protein that cooperates with other human RFX members.** *J Biol Chem* 2002, **277:**836-842.

29. Kistler WS, Horvath GC, Kistler MK: **RFX2 is a candidate downstream amplifier of A-MYB regulation in mouse spermatogenesis.** *Bmc Dev Biol* 2009, **9**.

30. Schimenti JC, Bolcun-Filas E, Bannister LA, Barash A, Schimenti KJ, Hartford SA, Eppig JJ, Handel MA, Shen LS: **A-MYB (MYBL1) transcription factor is a master regulator of male meiosis.** *Development* 2011, **138:**3319-3330.

31. Latham KE, Litvin J, Orth JM, Patel B, Mettus R, Reddy EP: **Temporal patterns of A-myb and B-myb gene expression during testis development.** *Oncogene* 1996, **13:**1161-1168.

32. Escriva H, Rodriguez-Pena A, Vallejo CG: **Expression of mitochondrial genes and of the transcription factors involved in the biogenesis of mitochondria Tfam, NRF-1 and NRF-2, in rat liver, testis and brain.** *Biochimie* 1999, **81:**965-971.

33. Bonizzi G, Karin M: **The two NF-kappa B activation pathways and their role in innate and adaptive immunity.** *Trends Immunol* 2004, **25:**280-288.

34. Chen HM, Zhang P, Voso MT, Hohaus S, Gonzalez DA, Glass CK, Zhang DE, Tenen DG: **Neutrophils and monocytes express high levels of PU.1 (Spi-1) but not Spi-B.** *Blood* 1995, **85:**2918-2928.

35. Fisher RC, Scott EW: **Role of PU.1 in hematopoiesis.** *Stem Cells* 1998, **16:**25-37.

36. Michaud J, Scott HS, Escher R: **AML1 interconnected pathways of leukemogenesis.** *Cancer Invest* 2003, **21:**105-136.

37. Lichtinger M, Hoogenkamp M, Krysinska H, Ingram R, Bonifer C: **Chromatin regulation by RUNX1.** *Blood Cell Mol Dis* 2010, **44:**287-290.

38. Perrotti D, Melotti P, Skorski T, Casella I, Peschle C, Calabretta B: **Overexpression of the Zinc-Finger Protein Mzf1 Inhibits Hematopoietic Development from Embryonic Stem-Cells - Correlation with Negative Regulation of Cd34 and C-Myb Promoter Activity.** *Mol Cell Biol* 1995, **15:**6075-6087.

39. Gaboli M, Kotsi PA, Gurrieri C, Cattoretti G, Ronchetti S, Cordon-Cardo C, Broxmeyer HE, Hromas R, Pandolfi PP: **Mzf1 controls cell proliferation and tumorigenesis.** *Gene Dev* 2001, **15:**1625-1630.

40. Taniguchi T, Ogasawara K, Takaoka A, Tanaka N: **IRF family of transcription factors as regulators of host defense.** *Annu Rev Immunol* 2001, **19:**623-655.

41. Cereghini S: **Liver-enriched transcription factors and hepatocyte differentiation.** *Faseb J* 1996, **10:**267-282.

42. Ktistaki E, Talianidis I: **Modulation of hepatic gene expression by hepatocyte nuclear factor 1.** *Science* 1997, **277:**109-112.

43. Zhao CY, Dahlman-Wright K: **Liver X receptor in cholesterol metabolism.** *J Endocrinol* 2010, **204:**233-240.

44. Schrem H, Klempnauer J, Borlak J: **Liver-enriched transcription factors in liver function and development. Part I: The hepatocyte nuclear factor network and liver-specific gene expression.** *Pharmacol Rev* 2002, **54:**129-158.

45. Hayhurst GP, Lee YH, Lambert G, Ward JM, Gonzalez FJ: **Hepatocyte nuclear factor 4alpha (nuclear receptor 2A1) is essential for maintenance of hepatic gene expression and lipid homeostasis.** *Mol Cell Biol* 2001, **21:**1393-1403.

46. Odom DT, Zizlsperger N, Gordon DB, Bell GW, Rinaldi NJ, Murray HL, Volkert TL, Schreiber J, Rolfe PA, Gifford DK, et al: **Control of pancreas and liver gene expression by HNF transcription factors.** *Science* 2004, **303:**1378-1381.

47. Georgopoulos K, Moore DD, Derfler B: **Ikaros, an Early Lymphoid-Specific Transcription Factor and a Putative Mediator for T-Cell Commitment.** *Science* 1992, **258:**808-812.

48. Spear BT, Jin L, Ramasamy S, Dobierzewska A: **Transcriptional control in the mammalian liver: liver development, perinatal repression, and zonal gene regulation.** *Cell Mol Life Sci* 2006, **63:**2922-2938.

49. Duncan SA: **Mechanisms controlling early development of the liver.** *Mech Develop* 2003, **120:**19-33.

50. Peters JM, Hollingshead HE, Gonzalez FJ: **Role of peroxisome-proliferator-activated receptor beta/delta (PPAR beta/delta) in gastrointestinal tract function and disease.** *Clin Sci* 2008, **115:**107-127.

51. Drewes T, Senkel S, Holewa B, Ryffel GU: **Human hepatocyte nuclear factor 4 isoforms are encoded by distinct and differentially expressed genes.** *Mol Cell Biol* 1996, **16:**925-931.

52. Babeu JP, Darsigny M, Lussier CR, Boudreau F: **Hepatocyte nuclear factor 4 alpha contributes to an intestinal epithelial phenotype in vitro and plays a partial role in mouse intestinal epithelium differentiation.** *Am J Physiol-Gastr L* 2009, **297:**G124-G134.

53. Ahn SH, Shah YM, Inoue J, Morimura K, Kim I, Yim S, Lambert G, Kurotani R, Nagashima K, Gonzalez FJ, Inoue Y: **Hepatocyte nuclear factor 4 alpha in the intestinal epithelial cells protects against inflammatory bowel disease.** *Inflamm Bowel Dis* 2008, **14:**908-920.

54. Don J, Stelzer G: **The expanding family of CREB/CREM transcription factors that are involved with spermatogenesis.** *Mol Cell Endocrinol* 2002, **187:**115-124.

55. Walker WH, Habener JF: **Role of transcription factors CREB and CREM in cAMP-regulated transcription during spermatogenesis.** *Trends Endocrin Met* 1996, **7:**133-138.

56. Pardo PS, Mohamed JS, Lopez MA, Boriek AM: **Induction of Sirt1 by Mechanical Stretch of Skeletal Muscle through the Early Response Factor EGR1 Triggers an Antioxidative Response.** *J Biol Chem* 2011, **286:**2559-2566.

57. Maass AH, Grohe C, Kubisch C, Wollnik B, Vetter H, Neyses L: **Hormonal Induction of an Immediate-Early Gene Response in Myogenic Cell-Lines - a Paradigm for Heart Growth.** *Eur Heart J* 1995, **16:**12-14.

58. Saadane N, Alpert L, Chalifour LE: **Altered molecular response to adrenoreceptor-induced cardiac hypertrophy in Egr-1-deficient mice.** *Am J Physiol-Heart C* 2000, **278:**H796-H805.

59. Khachigian LM: **Early growth response-1 in cardiovascular pathobiology.** *Circ Res* 2006, **98:**186-191.

60. Reizis B, Arenzana TL, Smith-Raska MR: **Transcription factor Zfx controls BCR-induced proliferation and survival of B lymphocytes.** *Blood* 2009, **113:**5857-5867.

61. Schaffner W, Wang Y, Wimmer U, Lichtlen P, Inderbitzin D, Stieger B, Meier PJ, Hunziker L, Stallmach T, Forrer R, et al: **Metal-responsive transcription factor-1 (MTF-1) is essential for embryonic liver development and heavy metal detoxification in the adult liver.** *Faseb J* 2004, **18:**1071-1079.

62. Trauth K, Mutschler B, Jenkins NA, Gilbert DJ, Copeland NG, Klempnauer KH: **Mouse a-Myb Encodes a Transactivator and Is Expressed in Mitotically Active-Cells of the Developing Central-Nervous-System, Adult Testis and B-Lymphocytes.** *Embo J* 1994, **13:**5994-6005.

63. Ihle JN: **The Stat family in cytokine signaling.** *Curr Opin Cell Biol* 2001, **13:**211-217.

64. Manley NR, Gordon J, Bennett AR, Blackburn CC: **Gcm2 and Foxn1 mark early parathyroid- and thymus-specific domains in the developing third pharyngeal pouch.** *Mech Develop* 2001, **103:**141-143.

65. Coffer PJ, Burgering BM: **Forkhead-box transcription factors and their role in the immune system.** *Nat Rev Immunol* 2004, **4:**889-899.

66. Green MR, Monti S, Dalla-Favera R, Pasqualucci L, Walsh NC, Schmidt-Supprian M, Kutok JL, Rodig SJ, Neuberg DS, Rajewsky K, et al: **Signatures of murine B-cell development implicate Yy1 as a regulator of the germinal center-specific program.** *P Natl Acad Sci USA* 2011, **108:**2873-2878.

67. Sweetser MT, Hoey T, Sun YL, Weaver WM, Price GA, Wilson CB: **The roles of nuclear factor of activated T cells and Ying-Yang 1 in activation-induced expression of the interferon-gamma promoter in T cells.** *J Biol Chem* 1998, **273:**34775-34783.

68. Wen AY, Sakamoto KM, Miller LS: **The Role of the Transcription Factor CREB in Immune Function.** *J Immunol* 2010, **185:**6413-6419.

69. Gilchrist M, Thorsson V, Li B, Rust AG, Korb M, Kennedy K, Hai T, Bolouri H, Aderem A: **Systems biology approaches identify ATF3 as a negative regulator of Toll-like receptor 4.** *Nature* 2006, **441:**173-178.

70. Whitmore MM, Iparraguirre A, Kubelka L, Weninger W, Hai T, Williams BRG: **Negative regulation of TLR-Signaling pathways by activating transcription factor-3.** *J Immunol* 2007, **179:**3622-3630.

71. Hirose N, Maekawa T, Shinagawa T, Ishii S: **ATF-2 regulates lipopolysaccharide-induced transcription in macrophage cells.** *Biochem Bioph Res Co* 2009, **385:**72-77.

72. Sitkovsky M, Lukashev D: **Regulation of immune cells by local. tissue oxygen tension: Hif1 alpha and adenosine receptors.** *Nat Rev Immunol* 2005, **5:**712-721.

73. Scott EW, Simon MC, Anastasi J, Singh H: **Requirement of Transcription Factor Pu.1 in the Development of Multiple Hematopoietic Lineages.** *Science* 1994, **265:**1573-1577.

74. Nuez B, Michalovich D, Bygrave A, Ploemacher R, Grosveld F: **Defective Hematopoiesis in Fetal Liver Resulting from Inactivation of the Eklf Gene.** *Nature* 1995, **375:**316-318.

75. Kuo CT, Veselits ML, Leiden JM: **LKLF: A transcriptional regulator of single-positive T cell quiescence and survival.** *Science* 1997, **277:**1986-1990.

76. Vanden Heuvel GB, Brantley JG, Alcalay NI, Sharma M, Kemeny G, Warolin J, Ledford AW, Pinson DM: **Hepatomegaly in transgenic mice expressing the homeobox gene Cux-1.** *Mol Carcinog* 2005, **43:**18-30.

77. Bosse T, Fialkovich JJ, Piaseckyj CM, Beuling E, Broekman H, Grand RJ, Montgomery RK, Krasinski SD: **Gata4 and Hnf1 alpha are partially required for the expression of specific intestinal genes during development.** *Am J Physiol-Gastr L* 2007, **292:**G1302-G1314.

78. Modica S, Gofflot F, Murzilli S, D'Orazio A, Salvatore L, Pellegrini F, Nicolucci A, Tognoni G, Copetti M, Valanzano R, et al: **The Intestinal Nuclear Receptor Signature With Epithelial Localization Patterns and Expression Modulation in Tumors.** *Gastroenterology* 2010, **138:**636-U295.

79. Ogura Y, Suruga K, Takase S, Goda T: **Developmental changes of the expression of the genes regulated by retinoic acid in the small intestine of rats.** *Life Sci* 2005, **77:**2804-2813.

80. Sarge KD, Parksarge OK, Kirby JD, Mayo KE, Morimoto RI: **Expression of Heat-Shock Factor-2 in Mouse Testis - Potential Role as a Regulator of Heat-Shock Protein Gene-Expression during Spermatogenesis.** *Biol Reprod* 1994, **50:**1334-1343.

81. Boulogne B, Levacher C, Durand P, Habert R: **Retinoic acid receptors and retinoid X receptors in the rat testis during fetal and postnatal development: Immunolocalization and implication in the control of the number of gonocytes.** *Biol Reprod* 1999, **61:**1548-1557.

82. Gaemers IC, van Pelt AMM, van der Saag PT, Hoogerbrugge JW, Themmen APN, de Rooij DG: **Differential expression pattern of retinoid X receptors in adult murine testicular cells implies varying roles for these receptors in spermatogenesis.** *Biol Reprod* 1998, **58:**1351-1356.

83. Gloss B, Trost SU, Bluhm WF, Swanson EA, Clark R, Winkfein R, Janzen KM, Giles W, Chassande O, Samarut J, Dillmann WH: **Cardiac ion channel expression and contractile function in mice with deletion of thyroid hormone receptor alpha or beta.** *Endocrinology* 2001, **142:**544-550.

84. Santalucia T, Moreno H, Palacin M, Yacoub MH, Brand NJ, Zorzano A: **A novel functional co-operation between MyoD, MEF2 and TR alpha 1 is sufficient for the induction of GLUT4 gene transcription.** *J Mol Biol* 2001, **314:**195-204.

85. White P, Burton KA, Fowden AL, Dauncey MJ: **Developmental expression analysis of thyroid hormone receptor isoforms reveals new insights into their essential functions in cardiac and skeletal muscles.** *Faseb J* 2001, **15:**1367-1376.

86. Yu FS, Gothe S, Wikstrom L, Forrest D, Vennstrom B, Larsson L: **Effects of thyroid hormone receptor gene disruption on myosin isoform expression in mouse skeletal muscles.** *Am J Physiol-Reg I* 2000, **278:**R1545-R1554.

87. Solomon SS, Majumdar G, Martinez-Hernandez A, Raghow R: **A critical role of Sp1 transcription factor in regulating gene expression in response to insulin and other hormones.** *Life Sci* 2008, **83:**305-312.

88. Zhang XH, Li YJ, Yang JW, Mundel P, Liu YH: **Sp1 and Sp3 transcription factors synergistically regulate HGF receptor gene expression in kidney.** *Am J Physiol-Renal* 2003, **284:**F82-F94.

89. Hu MC, Mo R, Bhella S, Wilson CW, Chuang PT, Hui CC, Rosenblum ND: **GLI3-dependent transcriptional repression of Gli1, Gli2 and kidney patterning genes disrupts renal morphogenesis.** *Development* 2006, **133:**569-578.

90. Eagon PK, Elm MS, Stafford EA, Porter LE: **Androgen Receptor in Human Liver - Characterization and Quantitation in Normal and Diseased Liver.** *Hepatology* 1994, **19:**92-100.

91. Pabst O, Forster R, Lipp M, Engel H, Arnold HH: **NKX2.3 is required for MAdCAM-1 expression and homing of lymphocytes in spleen and mucosa-associated lymphoid tissue.** *Embo J* 2000, **19:**2015-2023.

92. Tarlinton D, Light A, Metcalf D, Harvey RP, Robb L: **Architectural defects in the spleens of Nkx2-3-deficient mice are intrinsic and associated with defects in both B cell maturation and T cell-dependent immune responses.** *J Immunol* 2003, **170:**4002-4010.

93. Klaewsongkram J, Yang Y, Golech S, Katz J, Kaestner KH, Weng NP: **Kruppel-Like factor 4 regulates B cell number and activation-induced B cell proliferation.** *J Immunol* 2007, **179:**4679-4684.

94. Yusuf I, Kharas MG, Chen J, Peralta RQ, Maruniak A, Sareen P, Yang VW, Kaestner KH, Fruman DA: **KLF4 is a FOXO target gene that suppresses B cell proliferation.** *Int Immunol* 2008, **20:**671-681.

95. Guan HF, Xie LK, Leithauser F, Flossbach L, Moller P, Wirth T, Ushmorov A: **KLF4 is a tumor suppressor in B-cell non-Hodgkin lymphoma and in classic Hodgkin lymphoma.** *Blood* 2010, **116:**1469-1478.

96. Nakahata S, Yamazaki S, Nakauchi H, Morishita K: **Downregulation of ZEB1 and overexpression of Smad7 contribute to resistance to TGF-beta 1-mediated growth suppression in adult T-cell leukemia/lymphoma.** *Oncogene* 2010, **29:**4157-4169.

97. Ellis AL, Wang ZX, Yu XM, Mertz JE: **Either ZEB1 or ZEB2/SIP1 Can Play a Central Role in Regulating the Epstein-Barr Virus Latent-Lytic Switch in a Cell-Type-Specific Manner.** *J Virol* 2010, **84:**6139-6152.

98. Hu HM, Kanda K, Zhang L, Boxer LM: **Activation of the c-myc p1 promoter in Burkitt's lymphoma by the hs3 immunoglobulin heavy-chain gene enhancer.** *Leukemia* 2007, **21:**747-753.

99. Hecht JL, Aster JC: **Molecular biology of Burkitt's lymphoma.** *J Clin Oncol* 2000, **18:**3707-3721.

100. Pajic A, Spitkovsky D, Christoph B, Kempkes B, Schuhmacher M, Staege MS, Brielmeier M, Ellwart J, Kohlhuber F, Bornkamm GW, et al: **Cell cycle activation by c-myc in a Burkitt lymphoma model cell line.** *Int J Cancer* 2000, **87:**787-793.

101. Cheng LL, Guo JF, Sun LG, Fu J, Barnes PF, Metzger D, Chambon P, Oshima RG, Amagai T, Su DM: **Postnatal Tissue-specific Disruption of Transcription Factor FoxN1 Triggers Acute Thymic Atrophy.** *J Biol Chem* 2010, **285:**5836-5847.

102. Nehls M, Kyewski B, Messerle M, Waldschutz R, Schuddekopf K, Smith AJH, Boehm T: **Two genetically separable steps in the differentiation of thymic epithelium.** *Science* 1996, **272:**886-889.

103. Schafer S, Anschlag J, Nettersheim D, Haas N, Pawig L, Schorle H: **The role of BLIMP1 and its putative downstream target TFAP2C in germ cell development and germ cell tumours.** *Int J Androl* 2011, **34:**E152-E159.

104. Kurimoto K, Yabuta Y, Ohinata Y, Shigeta M, Yamanaka K, Saitou M: **Complex genome-wide transcription dynamics orchestrated by Blimp1 for the specification of the germ cell lineage in mice.** *Gene Dev* 2008, **22:**1617-1635.

105. Schultz R, Suominen J, Varre T, Hakovirta H, Parvinen M, Toppari J, Pelto-Huikko M: **Expression of aryl hydrocarbon receptor and aryl hydrocarbon receptor nuclear translocator messenger ribonucleic acids and proteins in rat and human testis.** *Endocrinology* 2003, **144:**767-776.

767-776.
